# Diversity at the *HYP1* locus in potato cyst nematodes does not result from developmentally-programmed somatic mutations

**DOI:** 10.1371/journal.pone.0358232

**Published:** 2026-09-15

**Authors:** Vincent C. T. Hanlon, Unnati Sonawala, Cian A. A. Raza, Luisa Kalkert, George Harpum, Lukas A. Burkhardt, Johannes Helder, Sebastian Eves-van den Akker

**Affiliations:** 1 Crop Science Centre, Department of Plant Sciences, University of Cambridge, Cambridge, United Kingdom; 2 Institute of Microbiology, Department of Biology, ETH Zürich, Zürich, Switzerland; 3 Laboratory of Nematology, Department of Plant Sciences, Wageningen University, Wageningen, The Netherlands; Indian Agricultural Research Institute, INDIA

## Abstract

Most genetic diversity stems from spontaneous mutations, that is, errors in DNA repair or replication. But for dozens of organisms across the tree of life, mutations at specific loci are not spontaneous but developmentally programmed: effectively, some organisms edit their own DNA sequences. This is perhaps most common among pathogens and parasites, many of which use editing to diversify genes that produce important antigens. Plant-parasitic potato cyst nematodes are damaging agricultural pests that establish a lifelong feeding site inside the root of their host plant. We previously observed extensive diversity of rare alleles at *HYP1*, the most highly expressed gene that encodes a protein secreted by potato cyst nematodes during parasitism. Importantly, *HYP1* alleles differ from each other by complex, in-frame rearrangements of short repeated sequence motifs within a single exon. Combining several lines of evidence, we previously hypothesized that potato cyst nematodes use developmentally-programmed mutations, or editing, to diversify *HYP1* alleles in the soma. In the current work, we now test this hypothesis. We employ highly accurate long-read DNA sequencing of a simplified genetic system to identify potential rare edited alleles, we use a transgenic yeast system to describe large *de novo* mutations at *HYP1*, and we interpret our findings in light of key population genetic parameters as well as the genetic diversity surrounding *HYP1* and across the genome.

## Introduction

Many parasitic nematode genomes harbour little genetic diversity [1, 2]. This is in stark contrast to their free-living relatives, particularly outcrossing species, which often have extremely high levels of genetic diversity [3]. Such patterns can be explained by population genetics theory, which predicts that selectively neutral genetic diversity increases with the effective population size at equilibrium [4]. Whereas some free-living nematodes have large census population sizes and efficient dispersal (which increase effective population size; e.g., [5]), the profound dependence of parasitic nematodes on their host plant or animal can often limit dispersal and promote inbreeding, lowering effective population sizes, strengthening genetic drift, and accelerating the loss of genetic variation genome-wide [1].

Conversely, a broad range of organisms—particularly pathogens and parasites—use developmentally-programmed mutations, or “editing”, to locally increase genetic diversity at targeted loci in their own genome [6]. Many pathogens edit the DNA sequences of genes that produce key antigens to escape recognition by the host immune system. For example, the bacteria, protozoa, and fungi that cause syphilis, sleeping sickness, and a form of pneumonia in humans are all believed to perform such editing [6]. Likewise, extensive somatic editing diversifies antigen receptor genes in the immune systems of jawed vertebrates [7] and, separately, of jawless vertebrates [8]. However, no such editing has yet been confirmed among the parasites or pathogens of plants.

**Fig 1:**
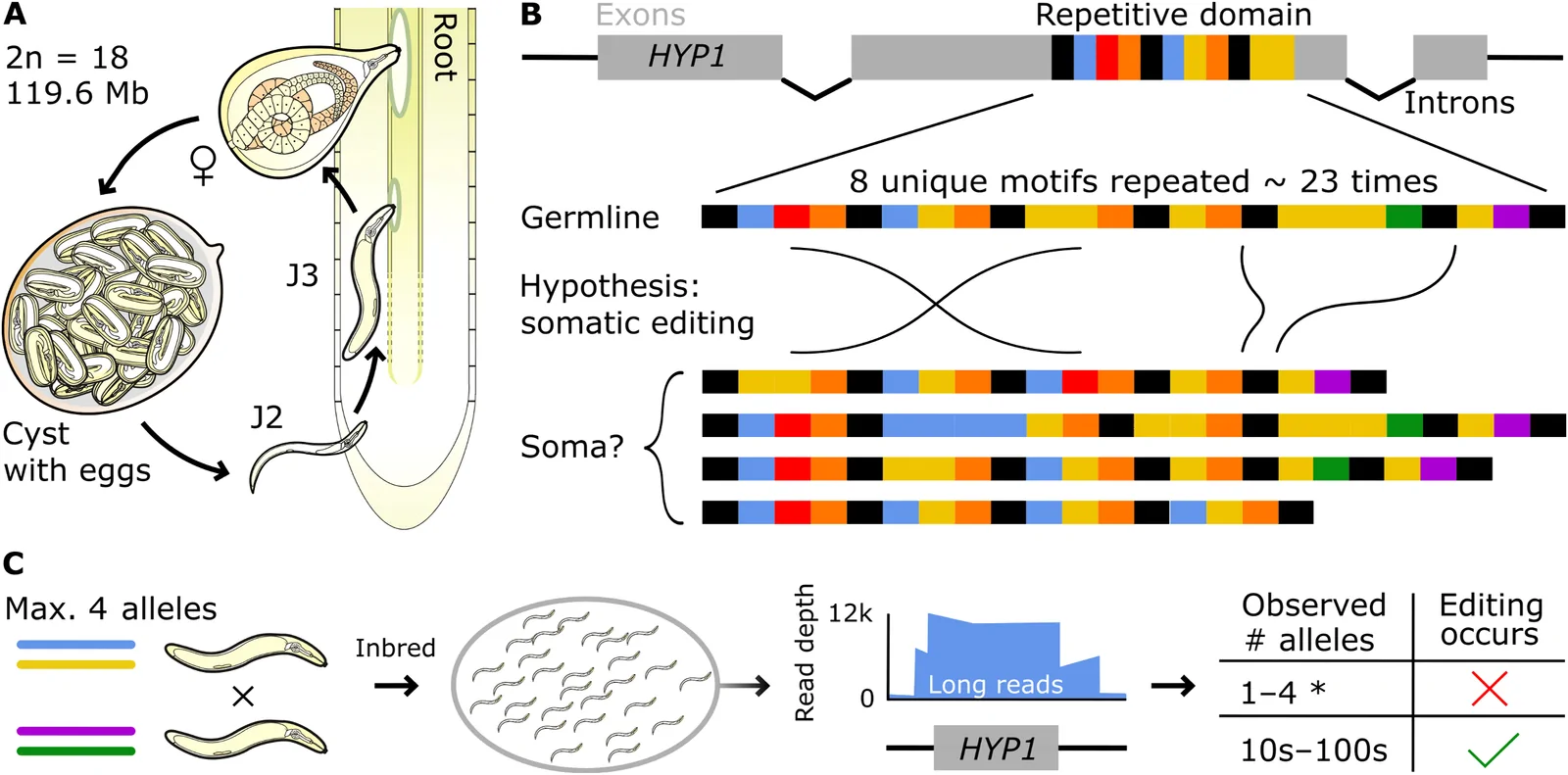
Experimental overview. (A) Simplified *Globodera* lifecycle with ploidy and genome size. J2, J3: stage 2, 3 juvenile. Males not shown. (B) Schematic showing how the somatic editing hypothesis could explain allelic diversity at *HYP1*. (C) Experimental approach for the *G. rostochiensis* inbred population derived from just 2 worms. Sharp changes in read depth around *GrHYP1* reflect the 4 Cas9 guide RNA cut sites. * Additional very rare alleles may also arise by spontaneous (non-developmentally-programmed) mutations.

We recently hypothesized that the allelic diversity observed at an important parasitism gene in potato cyst nematodes (*Globodera pallida* and *G.* *rostochiensis*) is the product of developmentally-programmed mutations in the soma [9], that is, somatic editing. The gene in question, *HYP1*, is required for successful parasitism of the host potato plants, during which it is the most highly expressed *G. pallida* gene encoding a secreted protein [10] (see Fig A in S1 Appendix on *HYP*-family genes). *HYP1* contains striking sequence diversity within the second exon: approximately 8 distinct sequence motifs (15 or 18 bp long) are repeated in complex permutations that form a repetitive domain of ~400 bp. Using long-read Oxford Nanopore Technologies sequencing (“ONT”) of native DNA and PacBio sequencing of amplicons, we previously observed that (i) *HYP1* alleles differ by complex, in-frame rearrangements of this repetitive domain, (ii) sequence reads from a single population contain many *HYP1* alleles, including many rare alleles, and (iii) PCR of *HYP1* from individual diploid worms yields more than two alleles [9]. To explain these results, we hypothesized that much like the pathogens of humans described above, *Globodera* nematodes might edit the DNA sequence of *HYP1* in some somatic cells, rearranging sequence motifs within the repetitive domain to diversify *HYP1* proteins and promote parasitism (Fig 1).

We now test the hypothesis that the observed allelic diversity at *HYP1* results from somatic editing, and we interpret our findings in the context of estimated effective population sizes as well as genetic diversity at the broader *HYP1* locus and across the genome.

## Results and discussion

### A simplified genetic system to investigate *HYP1* diversity

To distinguish rare edited somatic alleles from inherited germline variation, we examined an inbred *G. rostochiensis* population (Line 19) that descends exclusively from a single mating event [11], in which at most 4 germline *HYP1* alleles can have been inherited from the two diploid *G. rostochiensis* ancestors (“*GrHYP1*”; Fig 1C). This allowed us to design an unequivocal test of the editing hypothesis: somatic editing occurs only if pooled sequencing of unamplified DNA from this inbred population reveals tens or hundreds of rare *GrHYP1* alleles beyond the 4 that could have been inherited.

We obtained 12,268 reads covering the putatively edited repetitive domain within *GrHYP1* using Cas9-enriched long read ONT sequencing of native DNA. Most reads had reliable base quality scores at *GrHYP1*, with a mean of 28.6 (i.e., 0.14% error probability; SD 6.8; SD, standard deviation; Fig B in S1 Appendix). We used a reference-free approach to identify reads that contained *GrHYP1*, locate the repetitive domain, and parse each read into an interpretable *GrHYP1* allele consisting of a string of sequence motifs (15 or 18 bp long; see Materials and methods). Two common alleles accounted for 11,378 of the 11,417 reads that could be parsed in this way, with 39 reads apparently supporting rare alleles.

**Fig 2:**
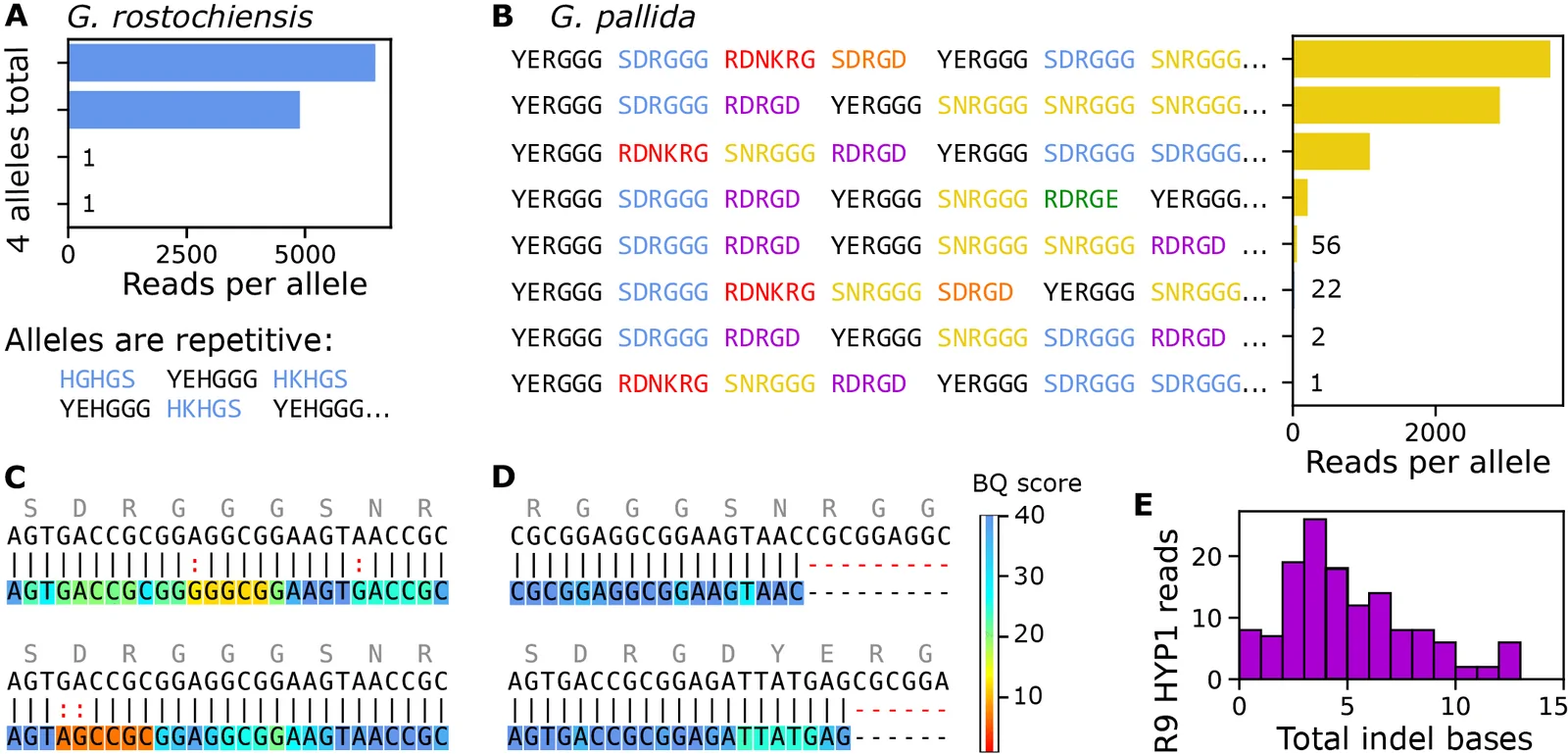
Observed *HYP1* repetitive domain alleles. (A) We see 2 common alleles and 2 putative rare alleles in the inbred population (see Table A in S1 Appendix for allele sequences). *G. rostochiensis* alleles have a simple tandem repeat structure. (B) In a laboratory-maintained field population of *G. pallida* we see 8 alleles total, which consist of complex rearrangements of sequence motifs (Table B in S1 Appendix). (C) Sequencing errors. For two putative rare alleles (bottom tracks; *G. pallida* R10 data), we show a portion of the alignment against the most similar ground-truth allele (top tracks). (D) Two putative rare alleles that passed manual examination, indicated by large indels embedded in good base quality (BQ) scores. (E) The old R9 reads match the 6 ground-truth *G. pallida* alleles, as shown by the cumulative length of all indels in alignments between each R9 read and the most similar ground-truth allele at the repetitive domain. Any edited alleles should contain rearrangements of 15 or 18 bp sequence motifs, and should therefore have 15 bp of indels at minimum (compare Fig E in S1 Appendix).

Under the somatic editing hypothesis, rare edited alleles should have large, complex rearrangements of sequence motifs relative to common germline alleles. We manually examined the 39 ONT reads bearing putative rare alleles by plotting the best alignment of each read against one of the two common alleles, coloured by base quality. This revealed that 37/39 reads were likely sequencing errors that differed from a common allele only by small variants embedded in lower-quality sequenced bases. The 2 remaining reads both had insertions embedded in good base qualities (33 and 132 bp; mean phred 29.8 and 36.6 for the repetitive domain), and may represent rare inherited germline variants, spontaneous mutations, or cryptic sequencing errors (Fig 2; see Figs C-D in S1 Appendix on the manual examination). Given that we found just 2–4 *GrHYP1* alleles among the 11,378 sequences from the inbred *G. rostochiensis* population, we concluded *GrHYP1* is not diversified by somatic editing.

### Re-examining *HYP1* diversity in *G. pallida*

In our previous work, *G. pallida HYP1* alleles (“*GpHYP1*”) were more diverse and more complex than *G. rostochiensis* [9]. We therefore decided to re-examine *GpHYP1* diversity in the same laboratory-maintained field population of *G. pallida* (Newton) studied previously, recognizing that ONT sequencing accuracy has improved substantially since the original publication (R9 vs R10 flow cell chemistry). After performing the same target-enriched sequencing and reference-free analysis as above, we obtained 7954 *G. pallida* reads with parsed *GpHYP1* alleles, including 3 common alleles supported by >1000 reads, 3 intermediate-frequency alleles supported by >20 reads, and at most 2 manually-validated rare alleles (Fig 2B-D; see Figs C-D in S1 Appendix on manual examination). The top 6 alleles were the same as those found in the 2024 study. However, our updated sequencing dataset contained far fewer rare alleles than previously observed, suggesting that developmentally-programmed somatic editing does not additionally diversify *GpHYP1* either.

To assess whether this reduced *GpHYP1* diversity might result from an unknown confounder, such as differences in growth conditions, we jointly analysed the old R9 chemistry ONT reads from our previous work [9] with ground truth allele sequences derived from our greatly improved R10 dataset. This showed that every old ONT *GpHYP1* read closely matched one of the top 6 alleles in the updated dataset, with at most 12 bp of indels flanked by low base qualities, indicating sequencing errors (Fig 2E). It may be that the structure of the *GpHYP1* repetitive domain somehow impairs ONT sequencing: even in the high-quality R10 dataset, we observed 15 bp deletion errors in 13 of the 7954 parsed reads, all in DNA sequences corresponding to “DRGDYERGGG” (Fig F in S1 Appendix). We additionally attribute the *GpHYP1* diversity previously observed in single-worm amplicon sequencing [9] to ubiquitous PCR template switching errors, which coincided with those observed from ONT sequencing and the hypothesized pattern of edited rearrangements.

### Yeast mutations simulate nematode *HYP1* alleles

**Fig 3:**
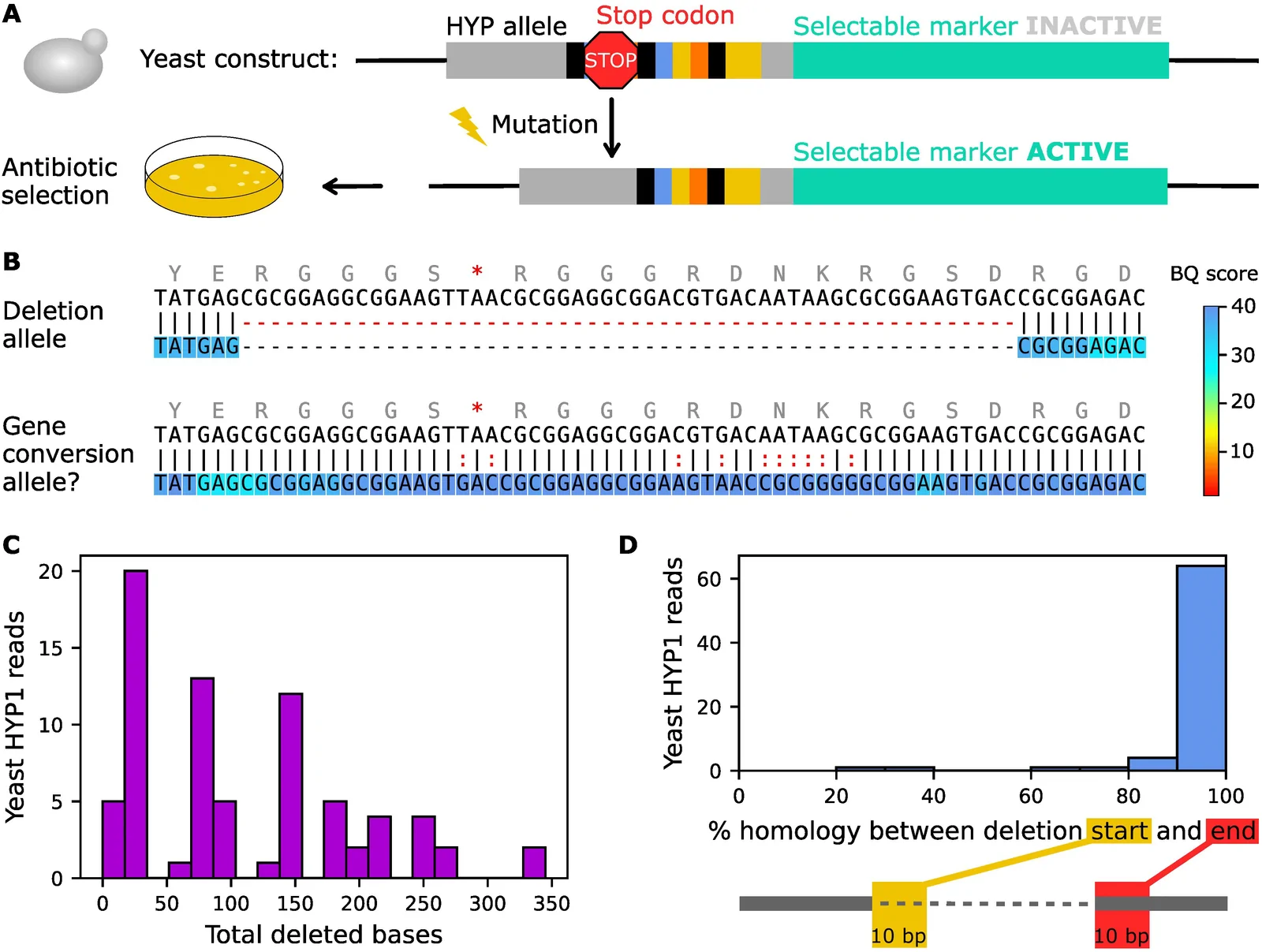
Mutations of the *GpHYP1* repetitive domain in transgenic yeast. (A) Simplified schematic of the experimental setup. (B) Partial alignments of two sequenced *GpHYP1* alleles in yeast (bottom tracks) against the allele from the original construct (top tracks), coloured by base quality (BQ). In the gene-conversion-like allele, DNA sequence corresponding to S*RGGGRDNKRG has been replaced with SDRGGGSNRGGG from downstream. Stop codons denoted “*”. (C) For each read, the total number of deleted bases relative to the original repetitive domain sequence. Most reads were dominated by one large deletion spanning the stop codon. (D) Nearly all deletions were embedded in sequence homology. Homology: fraction of matching bases. Only the largest deletion in each read (>10 bp) is considered.

Clearly, HYP1 diversity consists of germline alleles that arise by ordinary, spontaneous mutational processes rather than developmentally-programmed somatic editing. What mutations produce the complex sequence motif structure that defines HYP1? To address this question, we performed a reversion assay in a transgenic yeast system to enrich and sequence yeast colonies with mutated *GpHYP1*. Specifically, we fused the middle exon of the most common *GpHYP1* allele to the N-terminus of a gene conferring antibiotic resistance, placed a stop codon within a motif in the *GpHYP1* repetitive domain, and integrated this construct into the yeast genome (Fig 3). We pooled ~120 antibiotic-resistant yeast colonies for ONT sequencing (see Materials and methods; Supporting results in S1 Appendix) and obtained 76 reads bearing *GpHYP1* alleles. Of these, 72 deleted the stop codon along with adjacent sequences (mean 111 bp, SD 86 bp), 2 replaced the stop codon within probable gene conversion tracts, and 2 did not mutate either the stop codon or the *GpHYP1* sequence. Four of the reads additionally contained insertions of 18 bp or 51 bp. All observed mutations were located exclusively within the *GpHYP1* repetitive domain.

In general, the mutant alleles from yeast closely resembled nematode *GpHYP1* alleles. In at least 67 reads, the mutations preserved the *GpHYP1* motif structure: that is, the mutated repetitive domains consisted only of intact 15 or 18 bp sequence motifs seen in the nematode *GpHYP1* alleles, with the caveat that this finding is based on manual examination of individual ONT reads. Although the yeast mutant alleles were shorter (mean 349 bp, SD 89 bp) than natural nematode *GpHYP1* alleles (mean 452 bp, SD 17 bp), and although no mutant yeast allele was identical to a nematode *GpHYP1* allele, 5 mutant reads were highly similar to one of the manually-validated rare alleles in nematodes. These 5 reads differed from the rare allele only by ~12 single nucleotide variants (SNVs) but had the same underlying motif structure.

There are indications that most of the 74 mutant alleles may have arisen by homology-directed rearrangements of DNA sequence motifs in yeast. Strikingly, nearly all the deletions were embedded in microhomology, in the sense that their starts and ends contained the same sequence motifs (Fig 3D): all 4 rare alleles observed in nematodes likewise had mid-sized deletions or insertions (relative to the best-aligning common allele) embedded in microhomology. Moreover, for one or both of the gene-conversion-like alleles, the DNA sequence corresponding to YERGGG S*RGGG RDNKRG SDRGD appears to have been replaced with a homologous sequence present downstream in the same allele (YERGGG SDRGGG SNRGGG SDRGD; “*” denotes the stop codon). It seems plausible that erroneous homologous DNA repair of double-strand breaks, as reported for a similar yeast system [12], is the dominant mutational mechanism for the *GpHYP1* repetitive domain, given that the repair pathway involved is present in both yeast and nematodes (albeit with different protein machinery) [13]. However, we stress that alternate processes could also produce complex homology-directed rearrangements in *G. pallida*, for example, recurrent unequal crossing-over during meiosis.

### Characterising the genetic diversity surrounding *HYP1*

**Fig 4:**
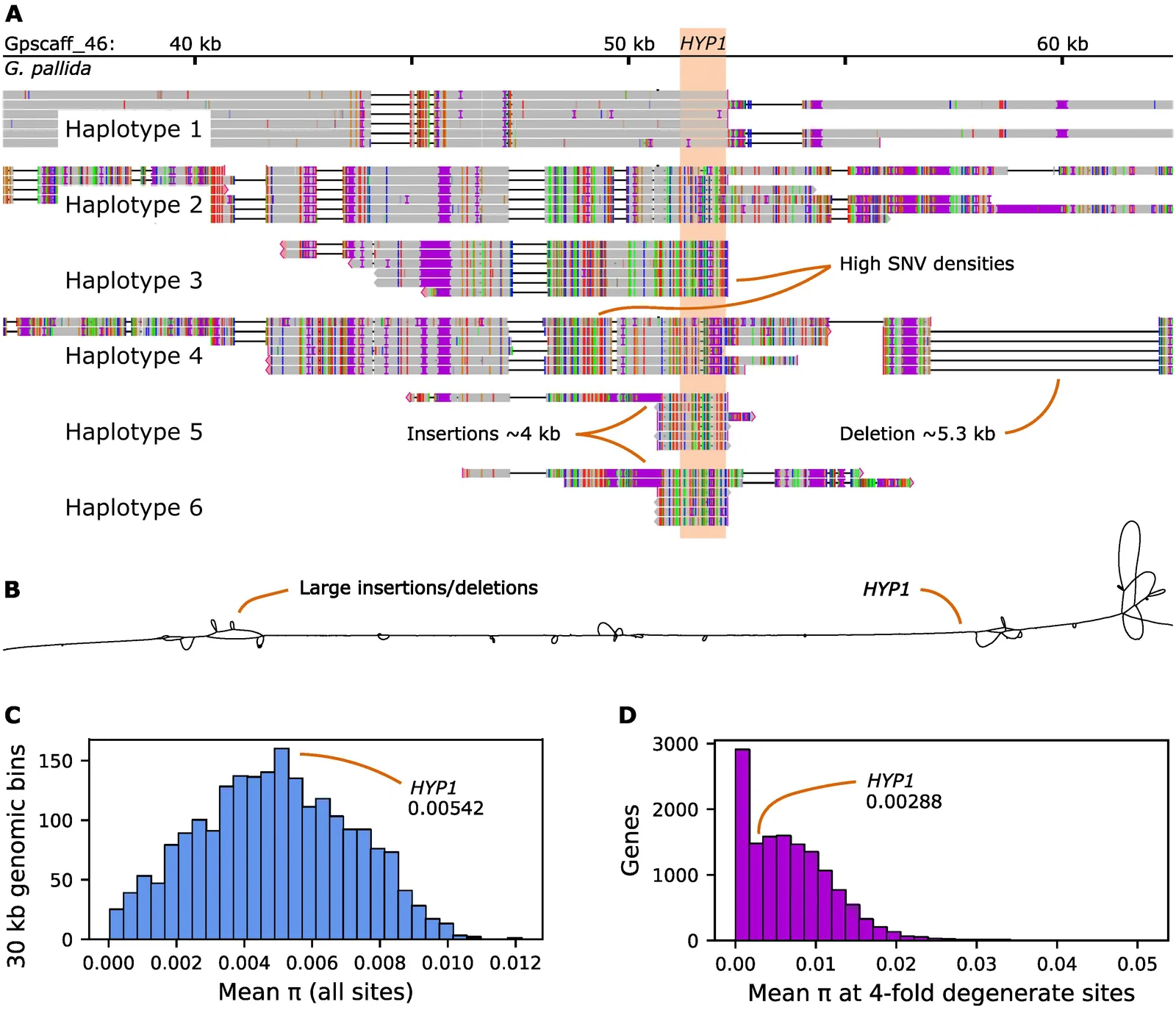
Genetic diversity of *GpHYP1*, the local region, and the genome. (A) Diversity among haplotypes near *GpHYP1*. In this genome browser snapshot, ONT reads from a population are grouped by the *GpHYP1* allele they bear. The sharp dropoff in coverage near *GpHYP1* is a consequence of Cas9 enrichment. Note that many reads align only partially because of reference bias. (B) Reference-free graph assembly of the top 4 haplotypes (1-4 above) for a region corresponding to approximately Gpscaff_46: 40-65 kb on the linear reference genome (as in A). (C) Mean nucleotide diversity (π) calculated on 30 kb bins using *G. pallida* short-read data on the linear reference genome (low-coverage genomic regions removed). Note that π here is approximately 2-fold lower than estimates from ONT long reads on the local graph assembly, perhaps due to reference bias. (D) Mean nucleotide diversity (π) calculated at 4-fold degenerate sites, binned by gene. π is zero-inflated, largely because short genes may contain few 4-fold degenerate sites.

The genomic region surrounding *GpHYP1* contains a striking diversity of SNVs and large insertions/deletions within a single population (Fig 4). The genomes of some nematode species are punctuated by “hyperdivergent regions” with dramatically elevated genetic diversity, which are often enriched for genes related to environmental or pathogen response [14, 15]. Investigating this possibility, we began by constructing ONT-based local assemblies for the haplotypes corresponding to the top 4 *GpHYP1* alleles (lengths 57.5–92.3 kb). Then, we combined the four haplotypes into a local graph assembly (see Materials and methods). Examining this graph assembly revealed that the four haplotypes are indeed interrupted by large, complex insertions/deletions (35 variants >1 kb) and are broadly collinear. Aligning the ONT reads to the graph assembly allowed us to call SNVs and estimate the mean nucleotide diversity at *GpHYP1* in our population based on the frequencies of the four haplotypes: this was 0.0137, corresponding to 1 SNV every 16 bp.

Although this is similar to hyperdivergent regions in species such as *Caenorhabditis briggsae* [15], hyperdivergent regions ultimately must have higher nucleotide diversity relative to the rest of the genome. We therefore aligned pre-existing short read DNA sequence data for a similar *G. pallida* (Newton) population [16] to the linear reference genome assembly [9], called SNVs, and calculated the mean nucleotide diversity both over 30 kb bins and also at 4-fold degenerate sites, which are thought to evolve nearly neutrally (Fig 4C-D; Materials and methods). Far from being unusual, this showed that the nucleotide diversity surrounding *GpHYP1* is typical of the *G. pallida* genome, suggesting that it is best explained by genome-wide neutral processes rather than local hyperdivergence.

For populations at equilibrium, high neutral nucleotide diversity corresponds to large effective population sizes (N_e_) [4]. Reported values of N_e_ for *G. pallida* and the related beet cyst nematode *Heterodera schachtii* were similar (64 and 85), despite being estimated under laboratory conditions and in a wild population, respectively [17,18]. Given the relationship π = 4N_e_μ and using 1.84 x 10^−9^ from the model nematode *C. elegans* for the mutation rate μ [19], we would expect neutral nucleotide diversity (π) to be near 5 x 10^−7^ at equilibrium. In fact, we find that mean nucleotide diversity genome-wide in our *G. pallida* (Newton) population is 7.46 x 10^−3^ at four-fold degenerate sites (Fig 4D): a discrepancy of four orders of magnitude.

Future research may illuminate the cause of this mismatch between the estimated effective population size and the observed nucleotide diversity. One possibility is that migration—which can greatly skew the π = 4N_e_μ relationship [20]—between *G. pallida* sub-populations could maintain relatively high genetic diversity despite low N_e_ within subpopulations. A second, related, possibility is that small N_e_ within subpopulations may reflect frequent inbreeding and other aspects of cyst nematode life history [18], but that long-term metapopulation N_e_ might be considerably larger. Because N_e_ determines the efficacy of natural selection and π relates to the amount of genetic variation available for adaptation, a better understanding of these parameters in cyst nematodes will be of great practical importance for assessing how quickly virulent strains are likely to arise that overcome genetic resistance in commercial potato varieties.

## Conclusions

In sum, we find that allelic diversity at the parasitism gene *HYP1* does not arise by somatic editing as hypothesized previously. Most likely, the mutations that produce *HYP1* alleles over evolutionary time are homology-directed rearrangements of sequence motifs. We describe elevated nucleotide diversity at *GpHYP1* and across the genome, which contrasts with the small effective population size reported for *G. pallida*. This work highlights the impact of recent progress in the accuracy of major DNA sequencing technologies, as well as the importance—and the challenges—of interrogating the genomes of non-model organisms.

## Materials and methods

### Growth conditions and sequencing

“Line 19” *G. rostochiensis* and *G. pallida* (Newton) nematodes were grown separately on Désirée potatoes in soil. Cysts were extracted by the Fenwick can method and cleaned by acetone floatation [21]. Cysts were either crushed between an aluminium block and a glass slide then floated on 50% iodixanol to obtain *G. rostochiensis* eggs (Supporting methods in S1 Appendix), or hatched in tomato root diffusate to obtain *G. pallida* J2s. We used phenol-chloroform to extract DNA and performed Cas9 enrichment as described previously [22], followed by ONT sequencing on the promethION R10 flow cell (Ligation sequencing DNA v14; see Table C for guide RNA sequences, as well as Supporting methods in S1 Appendix).

### Reference-free analysis of alleles

We selected all reads that contained a perfect match for either a known *HYP1* sequence motif or one of 6 arbitrarily-chosen 18mers from *HYP1* exon 2. We parsed the reads by replacing any DNA sequence that matched a known *HYP1* motif (up to edit distance 1) with its amino acid sequence. We discarded reads for which the repetitive domain could not be completely substituted into amino acid sequences in this way. *HYP1* alleles were considered identical if they were parsed into the same amino acid sequence, which allowed us to count the reads supporting each unique allele in a population.

For re-analysis of the R9 ONT reads from the 2024 study [9], we manually examined all 253 *GpHYP1-*containing reads with mean base quality greater than 16 (as in Figs C, D, and F in S1 Appendix).

### Transgenic yeast reversion assays

We assembled the TEF1 promoter, *GpHYP1* exon 2 interrupted by a stop codon, a rigid linker (“PAPAP”), and the antibiotic resistance gene AUR1-C into the pAbAi backbone (Takara) using a combination of overlap PCR, NEBuilder HiFi (New England Biolabs, “NEB”), and mutagenesis PCR, linearized it at *URA3* with BbsI-HF (NEB) and integrated it into the Y1Hgold yeast genome by lithium acetate heat shock [23]. We inoculated 40 mL of liquid synthetic dropout media without uracil (SD-URA), incubated for 3 days at 28°C, plated the yeast on SD-URA agar with 200 ng/mL Aureobasidin A (Supporting results in S1 Appendix), scraped off all colonies after 3 days at 28°C, washed twice with 0.9% NaCl and 0.01% TWEEN-20, and resuspended them in DNA/RNAshield (Zymo Research) for genomic DNA extraction and PCR-free ONT sequencing (Plasmidsaurus). We identified reads containing *GpHYP1* as described above for *G. pallida,* selected those with a mean base quality of at least 15 in the repetitive domain, and aligned all such reads against the expected (unmutated) sequence for manual examination (as in Figs C, D, and F in S1 Appendix).

### Graph assembly and variant analyses

Using *GpHYP1* ONT reads larger than 20 kb, we constructed allele-specific local assemblies either using flye v2.9.5-b1801 with --no-alt-contigs [24] or simply by choosing the two reads with greatest genomic span, aligning them to each other, and taking the consensus sequence. We polished the assemblies with racon v1.5.0 (github.com/isovic/racon) and then constructed a combined graph assembly with pggb v5134732 [25]. We aligned ONT reads to the graph using GraphAligner v1.0.17 [26] and called SNVs relative to the most common *GpHYP1* allele using vg v1.71.0 [27]. We aligned short reads (ENA accession ERR123954) to the linear reference assembly using NextGenMap v0.5.5 [28], called SNVs with freebayes v1.3.8 [29], and identified 4-fold degenerate sites with degenotate v1.2.4 [30] (see Supporting results, Supporting methods, and Figs G-H in S1 Appendix). Both variant callsets included SNVs (with QUAL>20) and invariant sites [31], used to estimate nucleotide diversity under the assumption that every read represents a distinct individual in pooled sequence data from thousands of nematodes.

## Supporting information

S1 Appendix Additional text, figures, and tables. This combined appendix describes the frequency of mutant alleles in yeast, SNV-calling adjustments for uncollapsed haplotypes, methods for egg extraction and Cas9-enrichment, and details of the variant calling approach for both the linear and graph assemblies. Additionally, it contains figures that describe known HYP-family genes, ONT base qualities, manual examination of HYP1 alleles, and SNV patterns related to uncollapsed haplotypes. Finally, it includes tables of consensus HYP1 allele sequences as well as Cas9 guide RNA sequences.
(PDF)
