# Supplementary material for: Diversity at the HYP1 locus in potato cyst nematodes does not result from developmentally-programmed somatic mutations
Source: PLoS One. 2026 Sep 15;21(9):e0358232. doi: 10.1371/journal.pone.0358232 (PMC13577501; doi:10.1371/journal.pone.0358232)
Supplement: Supplementary file 1 [file pone.0358232.s001.pdf]

S1 Appendix

Supporting results

HYP1 mutant frequency in a transgenic yeast system

We inoculated the SD-URA with one of two single yeast colonies: one with the *HYP1:AUR1-C* gene fusion interrupted by a stop codon in the *HYP1* repetitive domain (the treatment; see Materials and methods), and one with the same gene fusion without the stop codon (as a positive control). After ~3 days at 28°C, the OD600 was 0.892 for the treatment culture, and we plated 400 µL per plate onto 6 plates of SD agar -URA with 200 ng/mL of the antibiotic Aureobasidin A. For the control culture, OD600 was 0.876, and we diluted it to cell densities that were 1/20,000th and 1/1,000,000th of the treatment culture. The reason for this is that in the treatment culture, only yeast that has mutated the stop codon is expected to grow on the antibiotic, whereas in the control culture all yeast should grow. We spread 3 plates for each dilution of the control culture, and then incubated both treatment and control for 3 days at 28°C.

Approximate colony counts per plate were as follows: For the treatment plates, we counted 30, 23, 13, 24, 18, and 16 colonies. For the 1/20,000th positive control plates, we counted 147, 245, and 170 colonies. For the 1/1,000,000th positive control plates, we counted 7, 3, and 3 colonies. Combining these results, this suggests that approximately 1 in 200,000 yeast cells had a mutated stop codon.

Uncollapsed haplotypes & SNV calling

After mapping short reads (European Nucleotide Archive ERR123954) against the linear genome assembly for *G. pallida* (GenBank PRJNA702104), we observed a bimodal coverage distribution over 30 kb genomic bins, which strongly influenced the density of called SNVs (Fig G in S1 Appendix, below). This is problematic for calculating statistics such as nucleotide diversity,

because it is unclear whether (i) only the low-coverage regions are correctly represented in the assembly and contain reliable SNV calls, (ii) only the high-coverage regions are correctly represented, or (iii) both are correctly represented and the variation is biological (e.g., repetitive sequence or CNVs). *G. pallida* populations are known to be highly diverse, which complicates genome assembly efforts because diverged haplotypes for a single locus can be represented by several contigs, which are not always collapsed correctly to a single sequence in the haploid assembly. We suspected that the low-coverage bins were uncollapsed haplotypes. This was initially confirmed by an analysis in which we randomly selected 5 low-coverage and 5 high-coverage 30 kb genomic bins (>75X or < 75X depth of coverage). We extracted the FASTA sequences for the bins, removed them from the genome assembly, and then mapped the sequences back against the modified assembly using minimap2. The 5 low-coverage bins aligned to more sequences in the modified assembly than the high coverage bins (353 vs 285 alignments), the total alignments of low-coverage bins were longer (239 kb vs 149 kb), and the mapping quality scores were higher (12.4 vs 8.1). This is consistent with the low-coverage bins representing uncollapsed haplotypes, further confirmed by a more extensive read-mapping analysis (Fig H in S1 Appendix, below). Therefore, for SNV analyses such as calculating nucleotide diversity on the linear genome assembly, we used only variants from the high-coverage bins (>75X; same for the gene-level nucleotide diversity at 4-fold degenerate sites).

Supporting methods

Egg extraction by crushing cysts

For *G. rostochiensis,* we extracted eggs from cysts by crushing. Cysts were soaked in water overnight. We transferred a small batch of cysts to the trench on an aluminium block (75 x 24 x 6 mm, 0.5 mm trench: .sat file on DRYAD), removed excess water, and pressed down gently with a glass slide, smearing from side to side to break open the cysts. The cyst-egg mixture was transferred to a 50 mL Falcon tube by washing with 0.01% TWEEN-20. These crushing steps

were repeated several times to obtain more eggs. Then, we topped up the Falcon tube with 0.01% TWEEN-20 and pulse-vortexed on high to separate eggs from cysts (a small number of glass beads can be added to further improve separation). We centrifuged the cyst-egg mixture at 4000 x g for 2 min with brake set to 2, removed the supernatant, and then resuspended the pellet in the remaining ~1-5 mL. We then gently layered the cyst-egg mixture onto 5 mL 50% iodixanol in a 50 mL Falcon tube and centrifuged at 10000 x g for 2 min with brake set to 2. Cyst material and other debris settled to the bottom of the tube, and we collected the eggs floating on the iodixanol layer. It is important at this stage to make sure all pieces of cyst are removed from the egg mixture because they reduce the purity of extracted DNA. Finally, we washed the eggs twice with 0.01% TWEEN-20, pelleted them and removed the supernatant, and stored them at -80 C until DNA extraction.

Cas9-enriched DNA sequencing

We performed Cas9-enriched sequencing on two promethION R10 flow cells for the *G. rostochiensis* population and on one R10 flow cell for the *G. pallida* population. Library preparation and Cas9 enrichment was as previously described [22] (Ligation sequencing DNA v14), with guide RNAs as in Table C in S1 Appendix. Input DNA amounts ranged from 5 to 9 µg of HMW DNA, and the short fragment buffer was used during library preparation.

ONT personnel advised that the R10 flow cell is unsuitable for Cas9-enriched libraries because the flow cell cannot tolerate underloading, but in our hands this was not the case. For each sequencing experiment, we mixed two libraries: a Cas9-enriched library with the addition of approximately 0-30 fmol of an unenriched whole-genome library (1 ug input, prepared with the long fragment buffer) to avoid underloading. We washed and re-loaded each flow cell several times with different proportions of the unenriched library spiked in. We observed that the R10 flow cell performed best for a Cas9 enriched library when no unenriched library was spiked in,

and yielded more reads passing quality filters than the R9 flow cell had previously [22] (data not shown).

Variant calling: linear assembly

To calculate nucleotide diversity from short reads aligned to the linear *G. pallida* (Newton) genome assembly, we first called SNVs using freebayes as follows:

```
freebayes-parallel \
 <(fasta_generate_regions.py  Gpal_newton_newton.scaffolds.fa.fai 100000) 24 \
  -f Gpal_newton_newton.scaffolds.fa --report-monomorphic -F 0.01 -C 1 -g 1000 \
--pooled-continuous ERR123954.sorted.markdup.bam > ERR123954.incmonomorphic.vcf
```

We filtered the VCF to keep only SNVs and high-quality sites, without removing the invariant sites:

```
bcftools view ERR123954.incmonomorphic.vcf | \
bcftools filter -i '(TYPE="snp" && QUAL>20 && ALT!="*" || ALT=".")'  | \
bcftools view -Oz -H > ERR123954.incmonomorphic.filtered.vcf.gz
```

Variant calling: graph assembly

Calculating nucleotide on the graph assembly required calling SNVs relative to a designated reference path through the graph. We chose haplotype 1, corresponding to the most common *G. pallida HYP1* allele.

First, we aligned the Cas9-enriched ONT reads to the graph assembly. Note that this coverage drops off steeply away from *HYP1* in this dataset, so it is only suitable for estimating nucleotide diversity directly at *HYP1*.

```
GraphAligner -x vg -b 500 -g g1-4.fasta.3d73c94.11fba48.8088a73.smooth.final.gfa \
-f gpal_newt_mar2026.fastq -a gpal_newt_mar2026.local_graph.gaf -t 12
```

We ran subsequent commands from the pggb singularity container (github.com/pangenome/pggb). First we converted our graph assembly from .gfa format to .vg format:

```
singularity run -B pggb/data:/data pggb_latest.sif \
vg convert -g g1-4.fasta.3d73c94.11fba48.8088a73.smooth.final.gfa -p  \
> g1-4.fasta.3d73c94.11fba48.8088a73.smooth.final.vg
```

Then we called snarls on the graph and created a pack file:

```
singularity run -B pggb/data:/data pggb_latest.sif \
vg snarls g1-4.fasta.3d73c94.11fba48.8088a73.smooth.final.vg > graph.snarls
```

```
singularity run -B pggb/data:/data pggb_latest.sif \
vg pack -x g1-4.fasta.3d73c94.11fba48.8088a73.smooth.final.vg \
-a gpal_newt_mar2026.local_graph.gaf -t 12  -o gpal_newt_mar2026.local_graph.pack
```

Next, we called SNVs relative in linearized coordinates relative to haplotype 1:

```
singularity run -B pggb/data:/data pggb_latest.sif \
vg call g1-4.fasta.3d73c94.11fba48.8088a73.smooth.final.vg \
-k gpal_newt_mar2026.local_graph.pack -r graph.snarls -a \
-p 'Globodera_pallida_newton_hap1#1#HYP1#0' -t 12 > gpal_newt_mar2026.local_graph.vcf
```

This VCF file does not contain invariant sites, which are needed to calculate nucleotide diversity correctly. We added the invariant sites back in by first creating a mock all-sites VCF from the FASTA sequence for haplotype 1 and then merging it into the variant-only VCF with bcftools. The result was a VCF file of variant and invariant sites, equivalent to the one created for the linear assembly, above. We filtered the VCF to include only SNVs and good-quality sites as above, and then used it to estimate nucleotide diversity using our custom script (github.com/vincent-hanlon/hyp-globodera).

Supporting figures

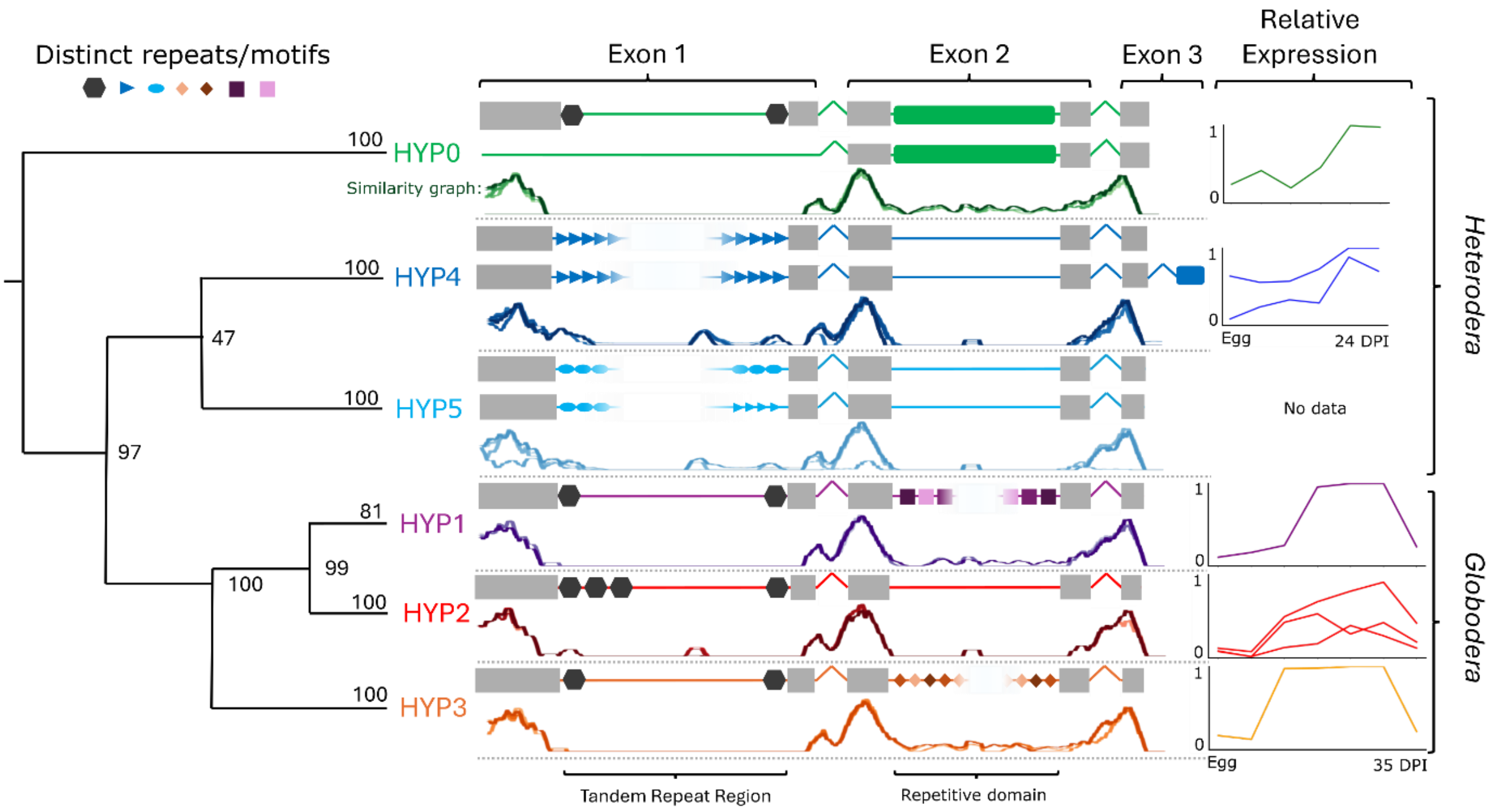

Figure A. Overview & phylogeny of *HYP*-family genes. *HYP1* is the most variable member of a 3-gene-family in *Globodera* and the subject of this work. *HYP1* and *HYP3* are single-copy genes with similarly-structured repetitive domains, but there are 2-3 *HYP2* genes and they have no repetitive domain (denoted by a straight line). *HYP*-family homologs exist in *Heterodera* as well, either with an invariant sequence in the repetitive domain (*HYP0)* or with no repetitive domain *(HYP4* and *HYP5).* Upstream, in Exon 1, some genes have a tandem repeat region that is similar to the repetitive domain but generally less variable. The phylogeny (MUSCLE, IQTree2) was constructed from *HYP*-family genes obtained by a BLAST search of genomes from six *Heterodera* and *Globodera* species. The similarity graph is a smoothed conservation score, which shows the fraction of matching residues in each column of the multiple sequence alignment. The relative expression track shows the percentile of each gene's expression relative to all other genes in the nematode at different life stages (i.e., 1 corresponds to the 100th percentile, meaning it is the most highly expressed gene at a time point). DPI: days post infection.

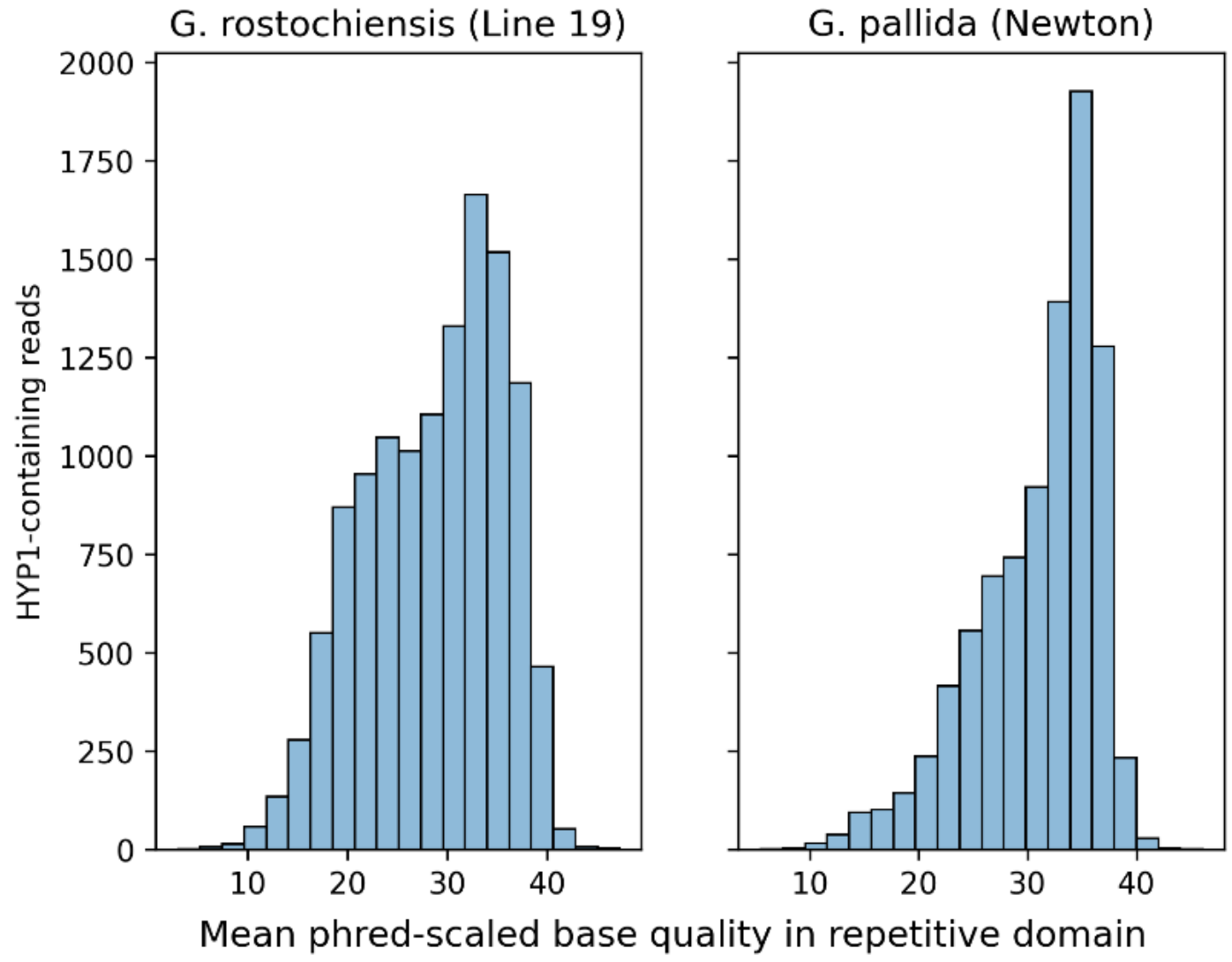

Figure B. Mean base quality of *HYP1*-containing reads (including reads that could not be parsed).

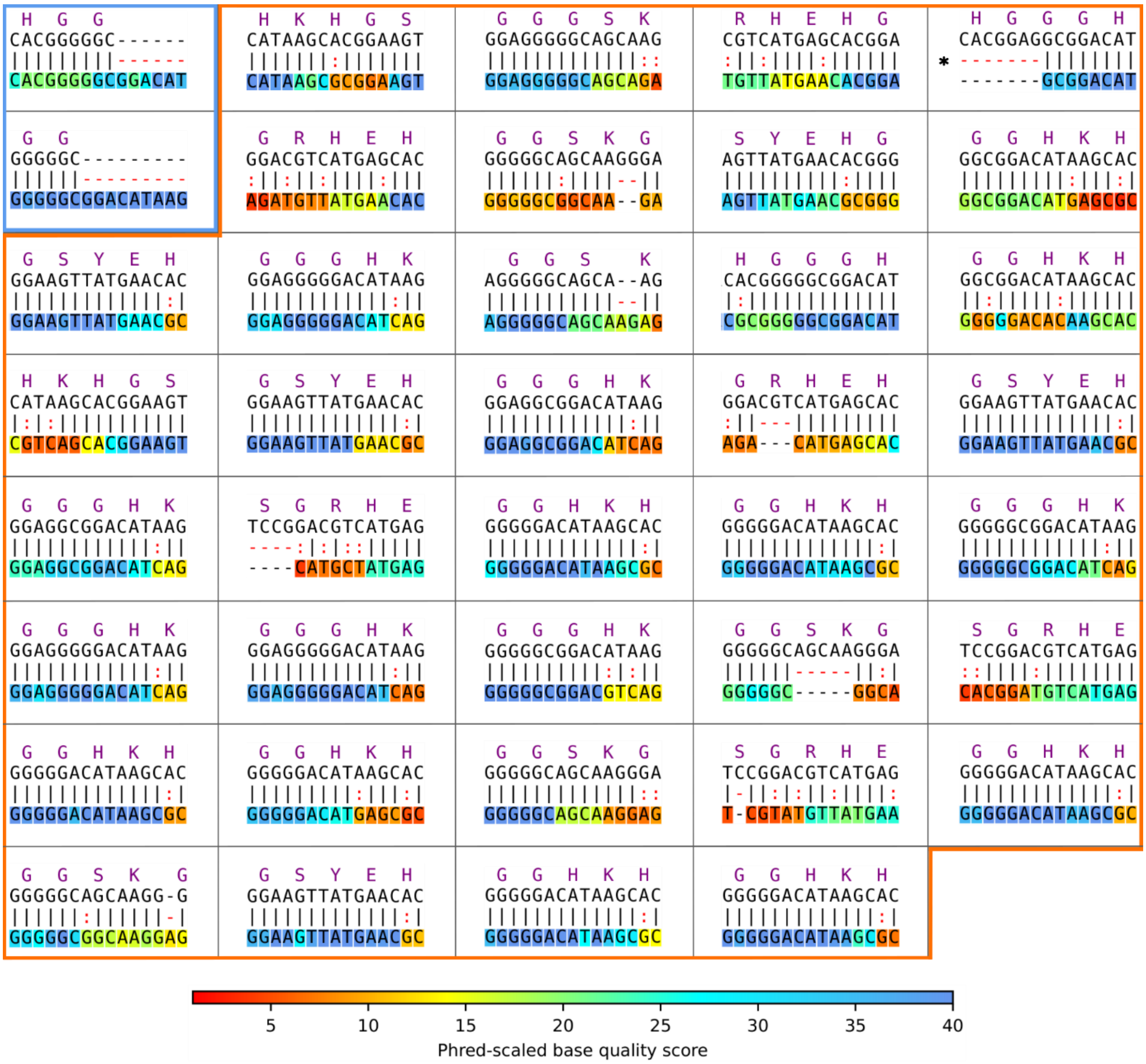

Figure C. Composite overview of manual examination of 39 putative rare alleles. We show the 39 parsed *HYP1*-containing reads from the *G. rostochiensis* inbred population (bottom; coloured by base quality), each aligned against the best-matching common allele from the same population (top; 2 common alleles total). Each alignment is ~400 bp long, but we show only the 15 bp window with the greatest number of mismatches and indels, within the *HYP1* repetitive domain. The 37 reads that are likely sequencing errors are outlined in orange, and the 2 reads that may represent real rare insertion alleles (length 33 and 99 bp, respectively) are outlined in blue. Because no quality filters were applied (so as not to overlook any informative reads) it is not surprising that nearly all putative rare alleles are sequencing errors. Note that although the read denoted (*) has base qualities >30 near the apparent deletion, further

investigation revealed this was actually an artefact of an alignment step in the processing pipeline used to locate the *HYP1* repetitive domain. Full alignment files for manual examination are available on Dryad Digital Repository (doi.org/10.5061/dryad.1c59zw4cd).

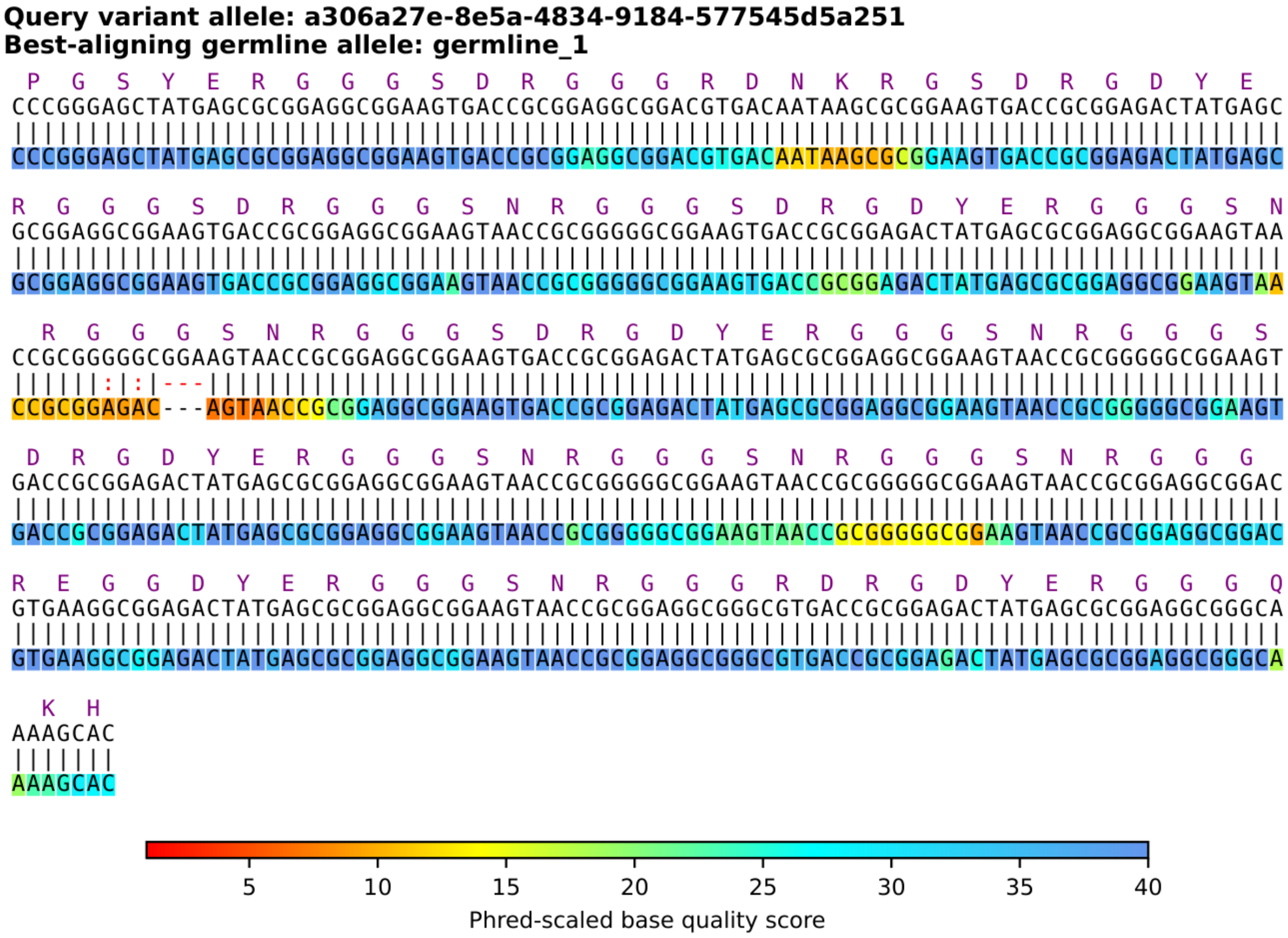

Figure D. Full-length manual examination of putative rare alleles. A potential variant *HYP1* R10 read from the laboratory-maintained *G. pallida* (Newton) field population is aligned against the best-matching common allele from the same population, showing the *HYP1* repetitive domain. We concluded that the 2 mismatched and 3 deleted bases are best explained by sequencing errors.

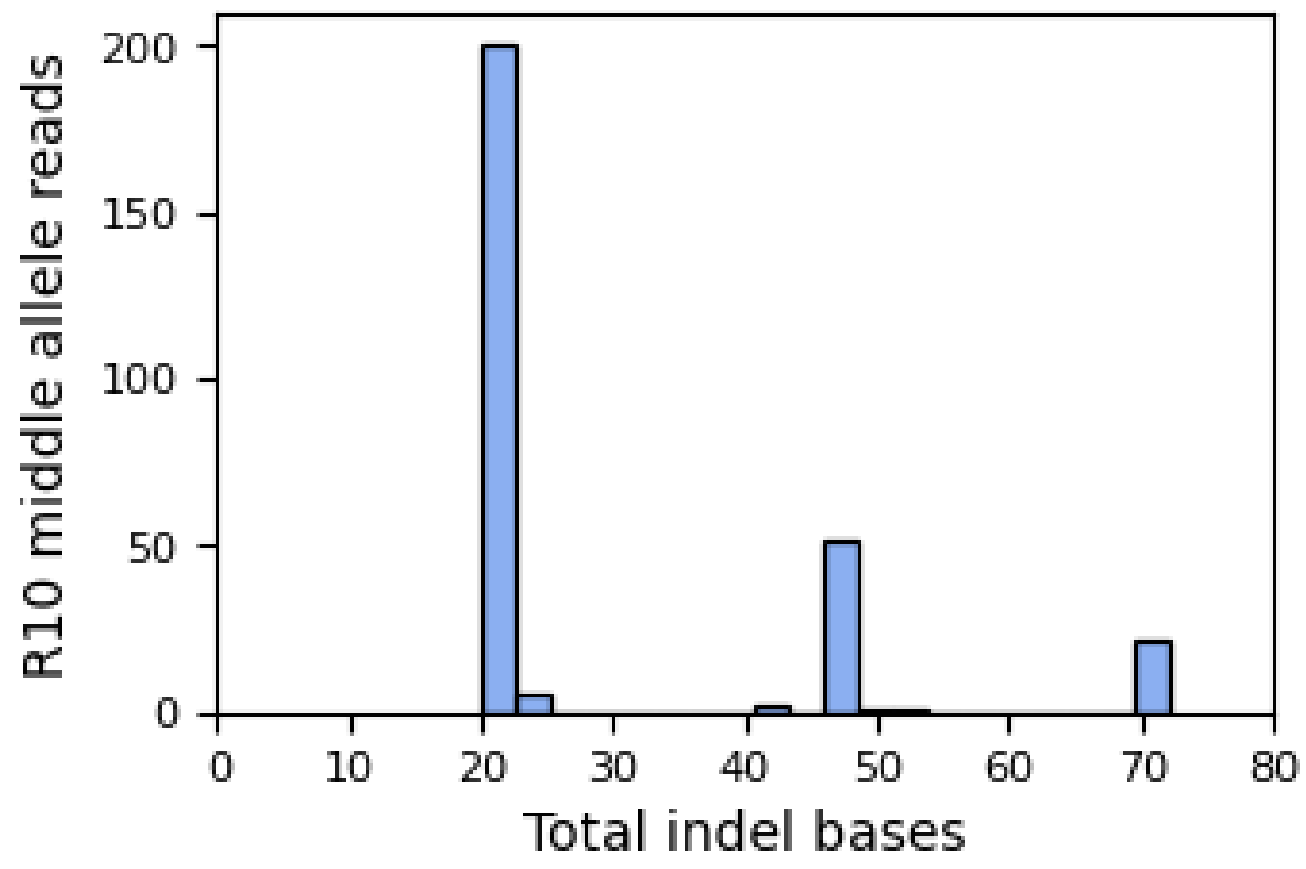

Figure E. A point of comparison for Fig 2E. Here, we align reads from the 4th, 5th, and 6th most common *HYP1* repetitive domain alleles in *G. pallida* (283 R10 reads total) against the most similar of the 3 most common alleles. Because the middle alleles differ from the top 3 alleles by rearrangements of 15 or 18 bp sequence motifs, the pattern is similar to what we would expect to see in rare edited alleles: all alignments contain >15 bp of indels. Note that indel sequence errors (rarer with R10 data than R9 data) are included in these counts.

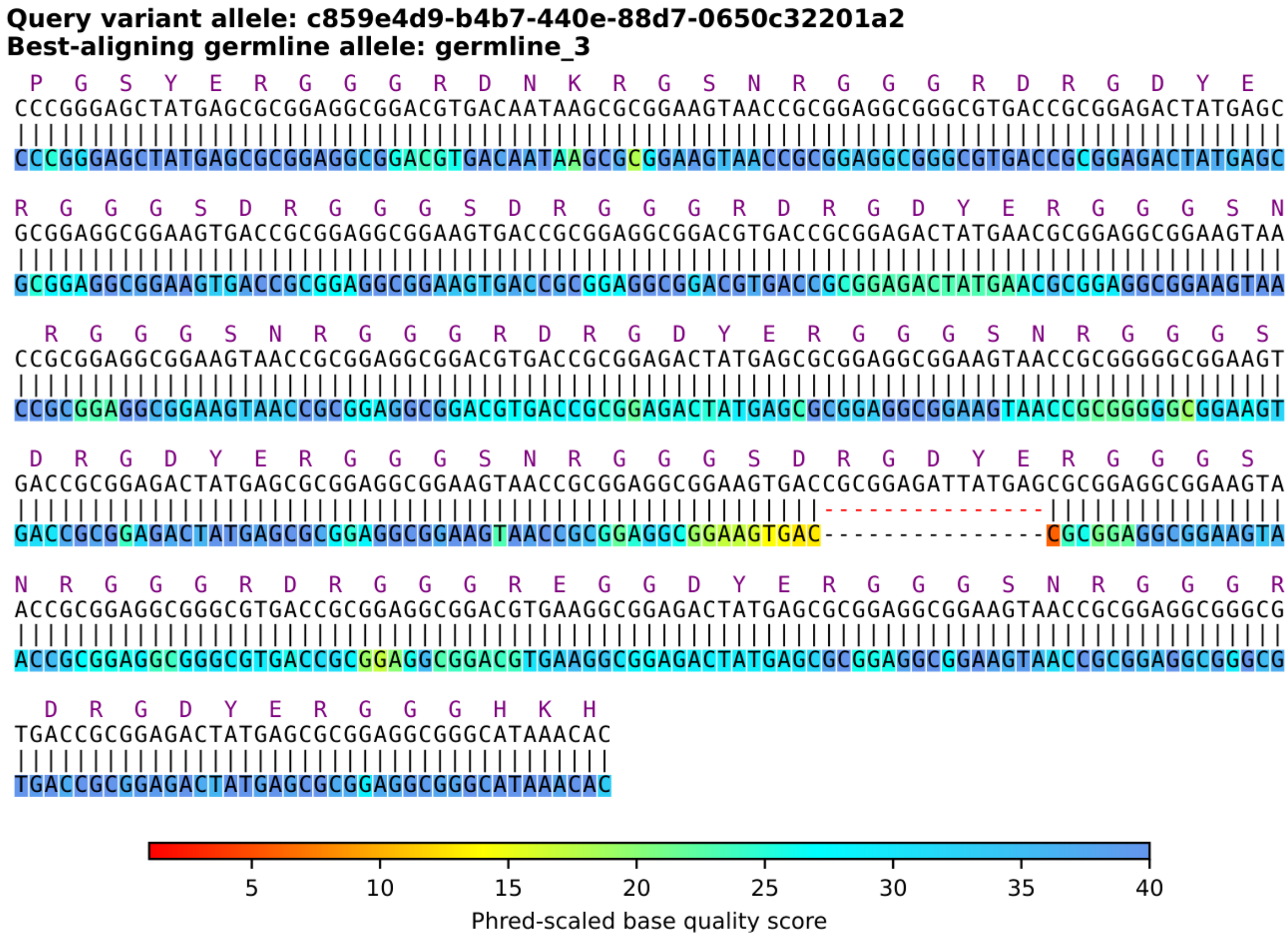

Figure F. A likely deletion error from ONT sequencing, shown as an alignment of the erroneous R10 read (bottom) against the best-aligning germline allele (top) at the *G. pallida HYP1* repetitive domain. We observed 15 such deletion errors in 13 reads, always at sequences that correspond to DRGDYERGGG and nearly always followed by a low-quality base.

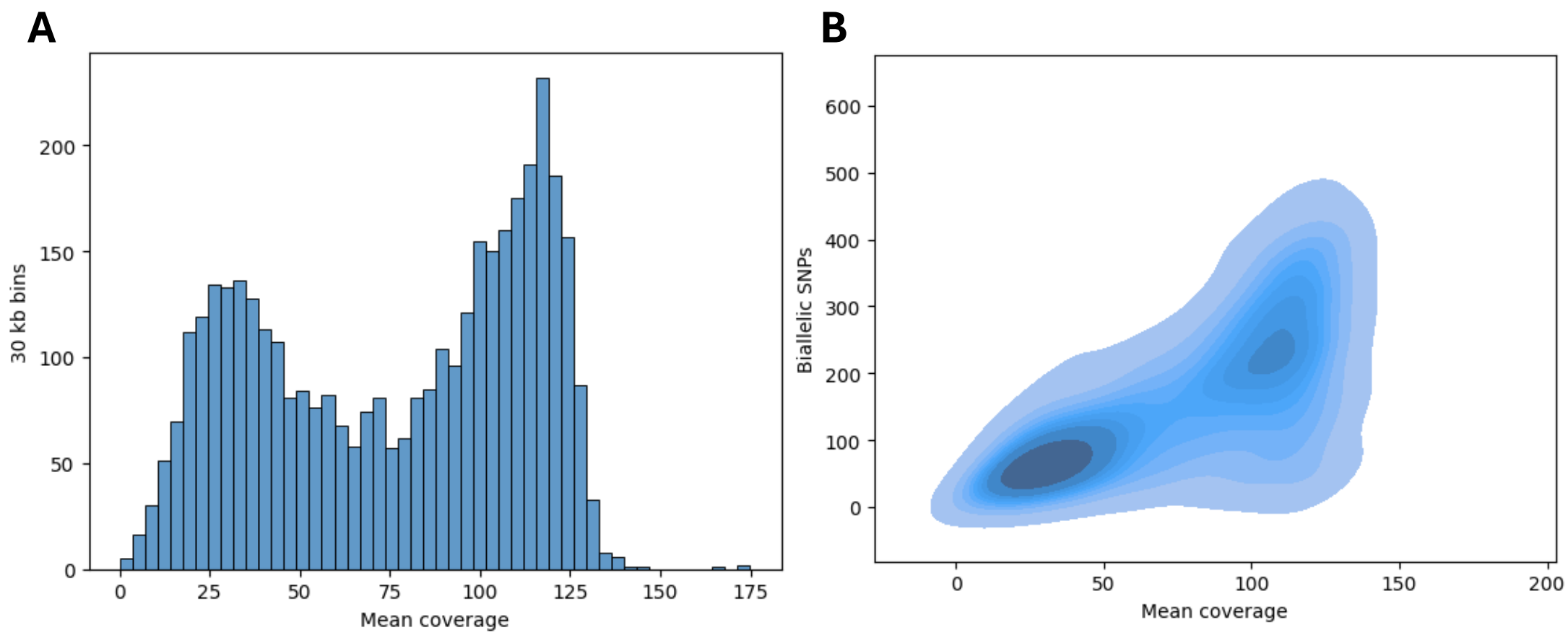

Figure G. Bimodal coverage and SNP density on the linear *G. pallida* (Newton) genome assembly (GenBank PRJNA702104). A., We divided the genome into 30 kb bins and used short reads to calculate the average depth of coverage across each bin. The resulting bimodal distribution could be explained by uncollapsed haplotypes in the assembly (Fig H in S1 Appendix, below). B., Low-coverage bins contain many fewer SNVs. This could be both an artefact of low coverage (less statistical power) and potentially a result of uncollapsed haplotypes (if alternate alleles simply map to the other haplotype).

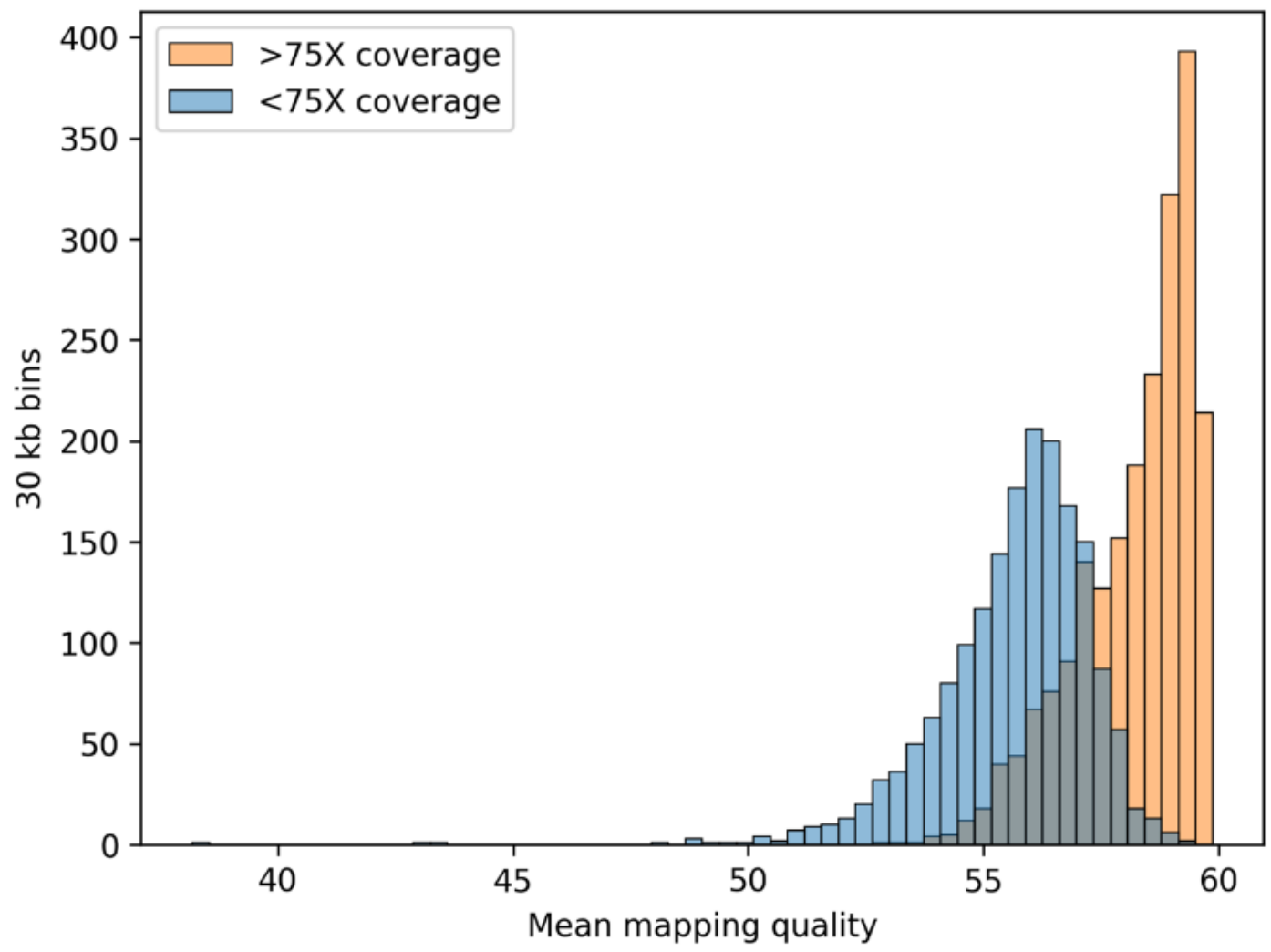

Figure H. Evidence of uncollapsed haplotypes in the linear *G. pallida* genome assembly. We predicted that if low-coverage regions are due to uncollapsed haplotypes, then short reads should have lower mapping qualities there because they would map reasonably well to 2+ contigs in the assembly (see also Fig G in and the Supporting methods in S1 Appendix).

Supporting tables

Table A. Consensus HYP1 repetitive domains alleles observed for *G. rostochiensis* (most common sequence provided where multiple reads exist).

Read count	Sequence
6486	GTGGCAAAAGATCCGGACGTCATGAGCACGGAGGCGGACATAAGCACGGAAGTTATGAACACGGGGGCGGACATAAGCACGGAAGTTATGAACACGGAGGGGGACATAAGCACGGAAGTTATGAACACGGAGGGGGACATAAGCACGGAAGTTATGAACACGGAGGGGGACATAAGCACGGAAGTTATGAACACGGAGGGGGACATAAGCACGGAAGTTATGAACACGGAGGGGGCAGCAAGGGAGAAAAGTACGA
4892	GTGGCAAAAGATCCGGACGTCATGAGCACGGAGGCGGACATAAGCACGGAAGTTATGAACACGGGGGCGGACATAAGCACGGAAGTTATGAACACGGAGGGGGACATAAGCACGGAAGTTATGAACACGGAGGGGGACATAAGCACGGAAGTTATGAACACGGAGGGGGACATAAGCACGGAAGTTATGAACACGGAGGGGGCAGCAAGGGAGAAAAGTACGA
1	GTGGCAAAAGATCCGGACGTCATGAGCACGGAGGCGGACATAAGCACGGAAGTTATGAACACGGGGGCGGACATAAGCACGGAAGTTATGAACACGGAGGGGGACATAAGCACGGAAGTTATGAACACGGAGGGGGACATAAGCACGGAAGTTATGAACACGGAGGGGGACATAAGCACGGAAGTTATGAACACGGAGGGGGACATAAGCACGGAAGTTATGAACACGGAGGGGGACATAAGCACGGAAGTTATGAACACGGAGGGGGCAGCAAGGGAGAAAAGTACGA
1	GTGGCAAAAGATCCGGACGTCATGAGCACGGAGGCGGACATAAGCACGGAAGTTATGAACACGGGGGCGGACATAAGCACGGAAGTTATGAACACGGAGGGGGACATAAGCACGGAAGTTATGAACACGGGGGCGGACATAAGCACGGAAGTTATGAACACGGAGGGGGACATAAGCACGGAAGTTATGAACACGGAGGGGGACATAAGCACGGAAGTTATGAACACGGAGGGGGACATAAGCACGGAAGTTATGAACACGGAGGGGGACATAAGCACGGAAGTTATGAACACGGAGGGGGACATAAGCACGGAAGTTATGAACACGGAGGGGGCAGCAAGGGAGAAAAGTACGA

Table B. Consensus HYP1 repetitive domains alleles observed for *G. pallida* (most common sequence provided where multiple reads exist).

Read count	Sequence
3607	GTCGTAAAAGACCCGGGAGCTATGAGCGCGGAGGCGGAAGTGACCGCGGAGGCGGACGTGACAATAAGCGCGGAAGTGACCGCGGAGACTATGAGCGCGGAGGCGGAAGTGACCGCGGAGGCGGAAGTAACCGCGGGGGCGGAAGTGACCGCGGAGACTATGAGCGCGGAGGCGGAAGTAACCGCGGGGGCGGAAGTAACCGCGGAGGCGGAAGTGACCGCGGAGACTATGAGCGCGGAGGCGGAAGTAACCGCGGGGGCGGAAGTGACCGCGGAGACTATGAGCGCGGAGGCGGAAGTAACCGCGGGGGCGGAAGTAACCGCGGGGGCGGAAGTAACCGCGGAGGCGGACGTGAAGGCGGAGACTATGAGCGCGGAGGCGGAAGTAACCGCGGAGGCGGGCGTGACCGCGGAGACTATGAGCGCGGAGGCGGGCAAAAGCACGGAGGTTATGA
2901	GTGGTAAAAGACCCGGGAGCTATGAGCGCGGAGGCGGAAGTGACCGCGGAGGCGGGCGTGACCGCGGAGACTATGAACGCGGAGGCGGAAGTAACCGCGGAGGCGGAAGTAACCGCGGAGGCGGAAGTAACCGCGGAGGCGGAAGTAACCGCGGAGGCGGACGTGACCGCGGAGACTATGAGCGCGGAGGCGGAAGTAACCGCGGGGGCGGAAGTGACCGCGGAGACTATGAGCGCGGAGGCGGAAGTGACCGCGGAGGCGGACGTGACCGCGGAGACTATGAGCGCGGAGGCGGAAGTAACCGCGGGGGCGGAAGTAACCGCGGAGGCGGACGTGAAGGCGGAGACTATGAGCGCGGAGGCGGAAGTAACCGCGGAGGCGGACGTGACCGCGGAGACTATGAGCGCGGAGGCGGGCATAAACACGGAGGTTATGA
1077	GTGGTAAAAGACCCGGGAGCTATGAGCGCGGAGGCGGACGTGACAATAAGCGCGGAAGTAACCGCGGAGGCGGGCGTGACCGCGGAGACTATGAGCGCGGAGGCGGAAGTGACCGCGGAGGCGGAAGTGACCGCGGAGGCGGACGTGACCGCGGAGACTATGAACGCGGAGGCGGAAGTAACCGCGGAGGCGGAAGTAACCGCGGAGGCGGACGTGACCGCGGAGACTATGAGCGCGGAGGCGGAAGTAACCGCGGGGGCGGAAGTGACCGCGGAGACTATGAGCGCGGAGGCGGAAGTAACCGCGGAGGCGGAAGTGACCGCGGAGATTATGAGCGCGGAGGCGGAAGTAACCGCGGAGGCGGGCGTGACCGCGGAGGCGGACGTGAAGGCGGAGACTATGAGCGCGGAGGCGGAAGTAACCGCGGAGGCGGGCGTGACCGCGGAGACTATGAGCGCGGAGGCGGGCATAAACACGGAGGTTATGA
205	GTGGTAAAAGACCCGGGAGCTATGAGCGCGGAGGCGGAAGTGACCGCGGAGGCGGACGTGACCGCGGAGACTATGAACGCGGAGGCGGAAGTAACCGCGGAGGCGGACGTGACCGCGGAGAGTATGAGCGCGGAGGCGGAAGTAACCGCGGAGGCGGAAGTAACCGCGGAGGCGGACGTGACCGCGGAGAGTATGAGCGCGGAGGCGGAAGTAACCGCGGGGGCGGAAGTGACCGCGGAGAGTATGAGCGCGGAGGCGGAAGTAACCGCGGAGGCGGACGTGAAGGCGGAGACTATGAGCGCGGAGGCGGGAGTAACCGCGGGGGCGGAAGTGACCGCGGAGAGTATGAGCGCGGAGGCGGAAGTAACCGCGGAGGCGGAAGTAACCGCGGAGGCGGACGTGACCGCGGAGACTATGAGCGCGGAGGCGGGCATAAGCACGGAGGTTATGA
56	GTGGTAAAAGACCCTGGAGCTATGAGCGCGGAGGCGGAAGTGACCGCGGAGGCGGACGTGACCGCGGAGACTATGAACGCGGAGGCGGAAGTAACCGCGGAGGCGGAAGTAACCGCGGAGGCGGACGTGACCGCGGAGACTATGAGCGCGGAGGCGGAAGTAACCGCGGGGGCGGAAGTGACCGCGGAGACTATGAGCGCGGAGGCGGAAGTAACCGCGGAGGCGGAAGTAACCGCGGAGGCGGAAGTGACCGCGGAGACTATGAGCGCGGAGGCGGAAGTAACCGCGGAGGCGGAAGTAACCGCGGAGGCGGACGTGAAGGCGGAGACTATGAGCGCGGAGGCGGAAGTAACCGCGGAGGCGGGCGTGACCGCGGAGACTATGAGCGCGGAGGCGGGCATAAGCACGGAGGTTATGA
22	GTGGTAAAAGACCCGGGAGCTATGAGCGCGGAGGCGGAAGTGACCGCGGAGGCGGACGTGACAATAAGCGCGGAAGTAACCGCGGAGGCGGAAGTGACCGCGGAGATTATGAGCGCGGAGGCGGAAGTAACCGCGGAGGCGGACGTGAAGGCGGAGACTATGAGCGCGGAGGCGGAAGTAACCGCGGAGGCGGGCGTGACCGCGGAGACTATGAGCGCGGAGGCGGAAGTAACCGCGGAGGCGGGCGTGACCGCGGAGACTATGAGCGCGGAGGCGGAAGTAACCGCGGGGGCGGAAGTGACCGCGGAGAGTATGAGCGCGGAGGCGGAAGTAACCGCGGAGGCGGAAGTGACCGCGGAGATTATGAGCGCGGAGGCGGAAGTAACCGCGGAGGCGGGCGTGACCGCGGAGACTATGAGCGCGGAGGCGGGCATAAACACGGAGGTTATGA
2	GTGGTAAAAGACCTGGGAGCTATGAGCGTGGAGGTGGAAGTGACCGCGGAGGCGGACGTGACCGCGGAGACTATGAGCGCGGAGGCGGAAGTAACCGCGGAGGCGGAAGTGACCGCGGAGGCGGACGTGACCGCGGAGACTATGAGCGCGGAGGCGGAAGTAACCGCGGAGGCGGAAGTGACCGCGGAGATTATGAGCGCGGAGGCGGAAGTAACCGCGGGGGCGGAAGTAACCGCGGAGGCGGAAGTAACCGCGGAGGCGGACGTGAAGGCGGAGACTATGAGCGCGGAGGCGGAAGTAACCGCGGAGGCGGACGTGACCGCGGAGACTATGAGCGCGGAGGCGGGCATAAACACGGAGGTTATGA
1	GTGGTAAAAGACCCGGGAGCTATGAGCGCGGAGGCGGACGTGACAATAAGCGCGGAAGTAACCGCGGAGGCGGGCGTGACCGCGGAGACTATGAGCGCGGAGGCGGAAGTGACCGCGGAGGCGGAAGTGACCGCGGAGGCGGACGTGACCGCGGAGACTATGAACGCGGAGGCGGAAGTAACCGCGGAGGCGGAAGTAACCGCGGAGGCGGACGTGACCGCGGAGACTATGAGCGCGGAGGCGGAAGTAACCGCGGGGGCGGAAGTGACCGCGGAGACTATGAGCGCGGAGGCGGAAGTAACCGCGGAGGCGGAAGTGACCGCGGAGATTATGAGCGCGGAGGCGGGCGTGACCGCGGAGGCGGACGTGAAGGCGGAGACTATGAGCGCGGAGGCGGAAGTAACCGCGGAGGCGGGCGTGACCGCGGAGACTATGAGCGCGGAGGCGGGCATAAACACGGAGGTTATGA

Table C. Guide RNA sequences for Cas9 enrichment of *HYP1*.

Species	Sequence
pallida	AUUGUAUGAUUGUCAAAAUC
pallida	AAUCAAAUGACACAUUACAA
rostochiensis	GGUCAUUGCGCUUAAUGCGA
rostochiensis	GUUAUUAACGACAUUUGGAC
rostochiensis	AAUAACUCCCGGCGACGUCGCGG
rostochiensis	GUAUGUGCGCAACAAGGUUU
