# Supplementary material for: Diversity at the HYP1 locus in potato cyst nematodes does not result from developmentally-programmed somatic mutations
Source: PLoS One. 2026 Sep 15;21(9):e0358232. doi: 10.1371/journal.pone.0358232 (PMC13577501; doi:10.1371/journal.pone.0358232)
Supplement: Supplementary file 2 [file pone.0358232.s002.pdf]

August 27, 2026

PLOS
1875 Mission Street
Suite 103 #188
San Francisco, CA 94103
USA

Dear Dr. Dutta,

We are encouraged by your positive assessment of our manuscript, which we have revised according to your suggestions as well as those of the two reviewers.

Substantial improvements to the clarity, accuracy, and interpretation of our work, prompted by the reviewers' insightful commentary, are described in our point-by-point response to the reviews, below. Probably the most substantial changes were to the section of the Results and discussion which covers the transgenic yeast work: both reviewers asked for more detail here, and so we have added more analysis, better quantification, and a more balanced interpretation of the experiment. We have also altered the section headings, supporting information, figures, etc. to conform with the PLOS One formatting guidelines.

We would like to use the version of the Funding Statement we previously submitted: we confirm it is correct. Moreover, we agree that the "funders provided support in the form of salaries for authors VCTH and SEvdA, but did not have any additional role in the study design, data collection and analysis, decision to publish, or preparation of the manuscript. The specific roles of these authors are articulated in the 'author contributions' section."

We look forward to your comments on this improved version of our manuscript.

Sincerely,

Vincent Hanlon

Crop Science Centre
Department of Plant Sciences
University of Cambridge
Cambridge, UK
vcth2@cam.ac.uk

Response to reviews

Reviewer #1: The authors start from the hypothesis that Globodera might edit the DNA sequence of HYP1 in some somatic cells. They test this by attempting to distinguish rare edited somatic alleles from inherited germline variation, using Cas9-enriched long-read ONT sequencing of native DNA. If somatic editing were occurring, they would expect rare alleles carrying large, complex rearrangements of sequence motifs relative to the common germline alleles. Contrary to the hypothesis, they find only 2-4 GrHYP1 alleles among 11,378 sequences from an inbred G. rostochiensis population, and conclude that GrHYP1 is not diversified by somatic editing. They then ask the same question of G. pallida, where the updated dataset contains far fewer rare alleles than previously reported, and trace the earlier signal to nanopore basecalling error within the repetitive domain and to PCR template switching in single-worm amplicon data. Having excluded editing, they propose that the remaining germline diversity arises through error-prone homologous repair of double-strand breaks, and that genome-wide nucleotide diversity is sustained by migration between subpopulations despite small local effective population sizes.

I find the central conclusion convincing. The demonstration that two independent technical artefacts can each reproduce the exact pattern predicted by the hypothesis is valuable well beyond this system. I recommend this article for publication and I have some minor revision/questions:

We are glad to hear that our work was favourably received, and we thank the reviewer for their assessment and suggestions.

In lines 129-139, the authors ask what mutations produce the complex sequence motif that defines HYP1, and they use the transgenic yeast system to conclude that erroneous homologous DNA repair of double-strand breaks are the dominant mechanism. The yeast experiment establishes that these mechanisms can generate the observed motif architecture, but not that it is the mechanism operating in Globodera. In Fig. 3., the authors establish the original construct sequence as the baseline, not the natural allelic+ diversity in G. pallida populations. Could the authors comment on whether the mutations recovered in yeast share diagnostic features with the natural GpHYP1 alleles, for instance, microhomology at rearrangement junctions or comparable size and motif-boundary distributions? Some support of this kind, or an explicit statement of the limitation would strengthen this inference.

Great suggestions. We have now expanded our discussion of the transgenic yeast experiment, with much more detail comparing yeast and nematode *GpHYP1* alleles, as well as more detail on the microhomology observed. We also agree that we don't know

whether the hypothesized rearrangement mechanism in yeast is also acting on *GpHYP1* in the nematodes, and we now acknowledge this limitation.

In particular, we have organized the text so that one paragraph compares yeast and nematode *GpHYP1* alleles, and a second paragraph describes the evidence for homology-directed rearrangements:

In general, the mutant alleles from yeast closely resembled nematode GpHYP1 alleles. In at least 67 reads, the mutations preserved the GpHYP1 motif structure: that is, the mutated repetitive domains consisted only of intact 15 or 18 bp sequence motifs seen in the nematode GpHYP1 alleles, with the caveat that this finding is based on manual examination of individual ONT reads. Although the yeast mutant alleles were shorter (mean 349 bp, SD 89 bp) than the nematode GpHYP1 alleles (mean 452 bp, SD 17 bp), and although no mutant yeast allele was identical to a nematode GpHYP1 allele, 5 mutant reads were highly similar to one of the manually-validated rare alleles in nematodes. These 5 reads differed from the rare allele only by ~12 single nucleotide variants (SNVs) but had the same underlying motif structure.

*There are indications that most of the 74 mutant alleles may have arisen by homology-directed rearrangements of DNA sequence motifs in yeast. Strikingly, nearly all the deletions were embedded in microhomology, in the sense that their starts and ends contained the same sequence motifs (Fig 3D): all 4 rare alleles observed in nematodes likewise had mid-sized deletions or insertions (relative to the best-aligning common allele) embedded in microhomology. Moreover, for one or both of the gene-conversion-like alleles, the DNA sequence corresponding to YERGGG S*RGGG RDNKRG SDRGD appears to have been replaced with a homologous sequence present downstream in the same allele (YERGGG SDRGGG SNRGGG SDRGD; "*" denotes the stop codon). It seems plausible that erroneous homologous DNA repair of double-strand breaks, as reported for a similar yeast system [12], is the dominant mutational mechanism for the GpHYP1 repetitive domain, given that the repair pathway involved is present in both yeast and nematodes (albeit with different protein machinery) [13]. However, we stress that alternate processes could also produce complex homology-directed rearrangements in G. pallida, for example, recurrent unequal crossing-over during meiosis.*

Reviewer #2: The HYP family in PCN (potato cyst nematodes) refers to hyper-variable apoplastic effector proteins that play an essential role in how sedentary plant-parasitic nematodes establish compatible interactions with their host plants. Among HYP effectors, the HYP1 gene displays an extraordinary, unprecedented level of sequence and structural diversity, even among individual nematodes. In the submitted article, the authors tested the hypothesis that a developmentally programmed editing process exists in both species of PCN, G. rostochiensis and G. pallida. Although the results did not confirm the existence of such an editing process in plant-parasitic nematodes, they also explore other mechanisms that might explain how such a complex sequence motif structure could have arisen in the HYP1 gene.

The manuscript is well written, quite interesting (even if the main hypothesis was not validated) and I fully support its publication after the implementation of some minor comments below:

We appreciate the reviewer's positive appraisal of our work, and we thank them for their excellent suggestions for improvements, which we address below.

- Whilst I acknowledge that the authors present and discuss their results concisely, it seems to me that reading this article would benefit from both better structuring (subheadings) in the 'Results/Discussion' section and a review of what should be presented as supplementary data or within the main text. There is a great deal of supplementary data, and almost every main result or figure has a corresponding entry in the supplementary data, which readers are invited to consult. I suggest : i) providing clearer guidance to the reader regarding the supplementary information presented in the supplementary documents in relation to the figures ; ii) and also reorganizing some parts like adding the supplementary document entitled 'HYP1 mutations observed in a transgenic yeast system' – which contains important information – to the main text, and instead placing the paragraph in the results section (lines 116 to 128) relating to potential confounding effect in the supplementary materials (as this will not alter the main conclusion set out just before).

We thank the reviewer for this thorough assessment of the manuscript's organization. In response to these suggestions, we have made several changes, as follows:

- We added subheadings to the Results and Discussion
- We went through references to the Supporting information and removed/altered those that were not appropriate.
- We now usually provide a clarifying phrase about the SI whenever we refer to it in the main text, to guide the reader (for example, "*see Figs C-D in S1 Appendix on manual examination*")
- We have kept the Supporting information as one document called the S1 Appendix (rather than splitting it into a dozen separate items of SI, which can be

confusing), and we have provided a detailed caption for all items in this appendix which will be displayed in the main text (see below).

- We broke up the large Supporting results and discussion section on the yeast experiment as suggested. We moved the discussion/analysis parts to the corresponding main Results and discussion, and we moved the methods parts to the corresponding main Materials and methods. We left a small part (on counting yeast colonies) in the Supporting information, as we do not think it is quite as generally interesting.
- However, we did not move the paragraph on confounding effects to the Supporting information as suggested. The reason for this is that we feel it is very important to be clear that it was DNA sequencing errors in the previous dataset which led us to develop the editing hypothesis in the first place, and to demonstrate that an updated analysis of the previous dataset (leveraging what we have learned from the new dataset) also doesn't show editing.

S1 Appendix. Additional text, figures, and tables. *This appendix describes the frequency of mutant alleles in yeast, SNV-calling adjustments for uncollapsed haplotypes, methods for egg extraction and Cas9-enrichment, and details of the variant calling approach for both the linear and graph assemblies. Additionally, it contains figures that describe known HYP-family genes, ONT base qualities, manual examination of HYP1 alleles, and SNV patterns related to uncollapsed haplotypes. Finally, it includes tables of consensus HYP1 allele sequences as well as Cas9 guide RNA sequences.*

- Line 42-43 : Can you support by a reference the statement that free living nematodes have large census population sizes and efficient dispersal ? This was not studied in Cole & Viney, 2018 and I have the feeling that is may be an overinterpretation of the fact that free living nematodes are more aboundant in soils. I am not sure that in arable field a PPN species will not have a census population size as large of that of a free living nematode species.

This is a good point: we weren't necessarily thinking of free-living soil nematodes in arable fields, so much as marine nematodes and free-living *Caenorhabditis* etc., and so we agree that the picture should be somewhat more varied. However, quite a few papers describe hyperdiversity, effective population size, and dispersal in natural *Caenorhabditis* populations (e.g., doi.org/10.1002/bies.201500053, doi.org/10.1093/molbev/msl097, doi.org/10.1111/mec.12281), as well as some less complete work on dispersal in marine nematodes (doi.org/10.1186/1742-9994-10-1).

We now cite the first paper (Cutter 2015, doi.org/10.1002/bies.201500053) as an example for the population genetics of free-living nematodes, and we moderate the statement in the main text:

Whereas some free-living nematodes have large census population sizes and efficient dispersal (which increase effective population size; e.g., [5])

- Line 79 : as the M&M is at the end, please indicate in the Results the names of the nematode populations (ie line 19 and Newton) used in this study. Qualify the Newton population as a field population maintained in laboratory rather than a « non inbred » population (there is natural inbreeding reported in many G. pallida populations, don't think that Newton is an exception).

Excellent point—this was an oversight on our part. In trying to clearly distinguish Newton from L19 to the reader, we called them inbred and non-inbred. However, of course *G. pallida* (Newton) nematodes inbreed as well. We now call the Newton population "a laboratory-maintained field population" rather than "a non-inbred population". We also now mention the population names (Newton/Line 19) at first mention in the Results and discussion as recommended.

- Investigating mutations using the yeast system was quite interesting but if I am correct yeast is a good model with great functional conservation accross animals for nucleotide excision or mismatch repair but I don't know if this holds true also for recombinational mutations. May be a point of discussion or a paragraph to add in the introduction in relation to this part of the results.

We thank the reviewer for this comment. Homologous DNA repair and recombination are functionally conserved across yeast and nematodes, but they don't necessarily perform these tasks using homologous protein machinery. We now clarify this as part of our expanded discussion of the yeast experiment (requested by both reviewers). A particularly valuable aspect of this comment is that it motivated us to write down an alternate hypothesis for how the nematodes might have rearranged *GpHYP1* over evolutionary time to produce the alleles we see today:

*There are indications that most of the 74 mutant alleles may have arisen by homology-directed rearrangements of DNA sequence motifs in yeast. Strikingly, nearly all the deletions were embedded in microhomology, in the sense that their starts and ends contained the same sequence motifs (Fig 3D): all 4 rare alleles observed in nematodes likewise had mid-sized deletions or insertions (relative to the best-aligning common allele) embedded in microhomology. Moreover, for one or both of the gene-conversion-like alleles, the DNA sequence corresponding to YERGGG S*RGGG RDNKRG SDRGD appears to have been replaced with a homologous sequence present downstream in the same allele (YERGGG SDRGGG SNRGGG SDRGD; "*" denotes the stop codon). It seems plausible that erroneous homologous DNA repair of double-strand breaks, as*

reported for a similar yeast system [12], is the dominant mutational mechanism for the GpHYP1 repetitive domain, given that the repair pathway involved is present in both yeast and nematodes (albeit with different protein machinery) [13]. However, we stress that alternate processes could also produce complex homology-directed rearrangements in G. pallida, for example, recurrent unequal crossing-over during meiosis.

- Figure 4D : I have not understood the interest of Figure 4D and have not seen any corresponding text in lines 152-160.

The purpose here is really to anticipate a question that we think some readers will have. When we report high nucleotide diversity at *HYP1* or even across the genome, they may wonder if this is due to diversifying selection on *HYP1,* or even diversifying selection on most genes across a small genome (although that shouldn't be true). We believe it's important to show that nucleotide diversity is relatively high at "neutral" 4-fold degenerate sites as well, and that the diversity at *HYP1* is not exceptional (compared with other genes) even when only neutral sites are considered. We have added a phrase to the main text describing Fig 4D in context:

We [...] calculated the mean nucleotide diversity <u>both</u> over 30 kb bins <u>and also at 4-fold degenerate sites, which are thought to evolve nearly neutrally</u>

- Lines 161 - 179: I am not convinced of the value of this paragraph and I think it should be deleted. Indeed, it adds nothing new: even if the estimate of Ne differed from that already published for G. pallida, it would be impossible to compare them, as the value obtained by the authors is closely linked to the population's rearing conditions in the laboratory. Furthermore, as the authors themselves mention, their estimate of Ne could be overestimated, as many parameters of the ideal population, as defined in the Wright–Fisher model, are / can be biased (an ideal population is characterised by the absence of migration, mutation, selection and generational overlap, by equal sex ratios, a constant size from one generation to the next, and random mating).

In retrospect, we agree that we speculated too extensively based on the mismatch between the effective population size and the observed nucleotide diversity, and we completely acknowledge that our *G. pallida* (Newton) population is not an ideal population at equilibrium. However, we do believe that it's of interest to highlight the mismatch between the effective population size and the observed nucleotide diversity—both because it would be fascinating to know in what way *G. pallida* populations are <u>not</u> at equilibrium, and because both parameters are important for predicting how/whether nematodes could overcome new host resistance. We are also encouraged by the fact that the estimated Ne for *G. pallida* and *H. schachtii* is so

similar, even though one was measured in a lab in pots, and one was measured on a wild beet population on the beach. We would contend that that this shows that the rearing conditions do not invalidate our comparisons. We also note that our rearing conditions for *G. pallida* were quite similar to the study that estimated Ne: both grown on potato, in pots, under laboratory conditions.

We have re-written these two paragraphs extensively, and we hope this will be an acceptable compromise. First, we now focus on nucleotide diversity, rather than Ne, because it's what was actually observed in our study (and we agree it doesn't make sense to use the pi/Ne relationship to estimate Ne). Second, we have simplified the discussion of the causes of the Ne/pi discrepancy, and cast it much more explicitly in terms of avenues for future research rather than conclusions that can be definitively drawn from this study:

For populations at equilibrium, high neutral nucleotide diversity results from large effective population sizes (Ne) [4]. Reported values of Ne for G. pallida and the related beet cyst nematode Heterodera schachtii were similar (64 and 85), despite being estimated under laboratory conditions and in a wild population, respectively [16,17]. Given the relationship $\pi = 4Ne\mu$ and using 1.84 x 10-9 from the model nematode C. elegans for the mutation rate μ [15], we would expect neutral nucleotide diversity (π) to be near 5 x 10-7 at equilibrium. In fact, we find that mean nucleotide diversity genome-wide in our G. pallida (Newton) population is 7.46 x 10-3 at four-fold degenerate sites (Fig 4D): a discrepancy of four orders of magnitude.

Future research may illuminate the cause of this mismatch between the estimated effective population size and the observed nucleotide diversity. One possibility is that migration—which can greatly skew the $\pi = 4Ne\mu$ relationship [18]—between G. pallida sub-populations could maintain relatively high genetic diversity despite low Ne within subpopulations. A second, related, possibility is that small Ne within subpopulations may reflect frequent inbreeding and other aspects of cyst nematode life history [17], but that long-term metapopulation Ne might be considerably larger. Because Ne determines the efficacy of natural selection, and π relates to the amount of genetic variation available for adaptation, a better understanding of these parameters in cyst nematodes will be of great practical importance for assessing how quickly virulent strains are likely to arise that overcome genetic resistance in commercial potato varieties.

Finally, we have adjusted the sentence in the Conclusions that referred to these points:

We describe elevated rates of nucleotide diversity at GpHYP1 and across the genome, which contrasts with the small effective population size reported for G. pallida.
