# Supplementary material for: Diversity at the HYP1 locus in potato cyst nematodes does not result from developmentally-programmed somatic mutations
Source: PLoS One. 2026 Sep 15;21(9):e0358232. doi: 10.1371/journal.pone.0358232 (PMC13577501; doi:10.1371/journal.pone.0358232)
Supplement: Peer review history

# Peer review: Decision Letter 0

**DOI:** 10.1371/journal.pone.0358232.r001

**Authors:** Tushar Dutta

18 Aug 2026

Dear Dr. Hanlon,

Thank you for submitting your manuscript to PLOS One. After careful consideration, we feel that it has merit but does not fully meet PLOS One’s publication criteria as it currently stands. Therefore, we invite you to submit a revised version of the manuscript that addresses the points raised during the review process.

Please submit your revised manuscript by Sep 17 2026 11:59PM. If you will need more time than this to complete your revisions, please reply to this message or contact the journal office at plosone@plos.org. When you're ready to submit your revision, log on to https://www.editorialmanager.com/pone/ and select the 'Submissions Needing Revision' folder to locate your manuscript file.

- A letter that responds to each point raised by the academic editor and reviewer(s). You should upload this letter as a separate file labeled 'Response to Reviewers'.
- A marked-up copy of your manuscript that highlights changes made to the original version. You should upload this as a separate file labeled 'Revised Manuscript with Track Changes'.
- An unmarked version of your revised paper without tracked changes. You should upload this as a separate file labeled 'Manuscript'.

If you would like to make changes to your financial disclosure, please include your updated statement in your cover letter. Guidelines for resubmitting your figure files are available below the reviewer comments at the end of this letter.

If applicable, we recommend that you deposit your laboratory protocols in protocols.io to enhance the reproducibility of your results. Protocols.io assigns your protocol its own identifier (DOI) so that it can be cited independently in the future. For instructions see: https://journals.plos.org/plosone/s/submission-guidelines#loc-laboratory-protocols. Additionally, PLOS One offers an option for publishing peer-reviewed Lab Protocol articles, which describe protocols hosted on protocols.io. Read more information on sharing protocols at https://plos.org/protocols?utm_medium=editorial-email&utm_source=authorletters&utm_campaign=protocols.

As the corresponding author, your ORCID iD is verified in the submission system and will appear in the published article. PLOS supports the use of ORCID, and we encourage all coauthors to register for an ORCID iD and use it as well. Please encourage your coauthors to verify their ORCID iD within the submission system before final acceptance, as unverified ORCID iDs will not appear in the published article. *Only*  the individual author can complete the verification step; PLOS staff *cannot*  verify ORCID iDs on behalf of authors.

We look forward to receiving your revised manuscript.

Kind regards,

Tushar Kanti Dutta, Ph.D.

Academic Editor

PLOS One

Journal Requirements:

When submitting your revision, we need you to address these additional requirements.

1. Please ensure that your manuscript meets PLOS ONE's style requirements, including those for file naming. The PLOS ONE style templates can be found at

https://journals.plos.org/plosone/s/file?id=wjVg/PLOSOne_formatting_sample_main_body.pdf and

https://journals.plos.org/plosone/s/file?id=ba62/PLOSOne_formatting_sample_title_authors_affiliations.pdf

2. Thank you for stating the following in the Acknowledgments Section of your manuscript:

“V.C.T.H. is funded by a UK Horizons: Ember Fellowship (ARIA) and a Herchel Smith Postdoctoral Fellowship. Work on plant-parasitic nematodes at the University of Cambridge is supported by DEFRA licence 125034/359149/3, and funded by BBSRC grants BB/R011311/1, BB/S006397/1, BB/X006352/1, and BB/Y513246/1, a Leverhulme grant RPG-2023-001, and a UKRI FrontierResearch Grant EP/X024008/1, the Cambridge-Africa ALBORADA Research Fund, a School of Biological Sciences–Isaac Newton Trust Joint Seed Funding Award, and the Gatsby Charitable Foundation.”

We note that you have provided funding information that is not currently declared in your Funding Statement. However, funding information should not appear in the Acknowledgments section or other areas of your manuscript. We will only publish funding information present in the Funding Statement section of the online submission form.

Please remove any funding-related text from the manuscript and let us know how you would like to update your Funding Statement. Currently, your Funding Statement reads as follows:

“V.C.T.H. is funded by a UK Horizons: Ember Fellowship (ARIA; www.renaissancephilanthropy.org/uk-horizons/) and a Herchel Smith Postdoctoral Fellowship (www.herchelsmith.cam.ac.uk). Work on plant-parasitic nematodes at the University of Cambridge is supported by DEFRA licence 125034/359149/3 (www.gov.uk/government/organisations/department-for-environment-food-rural-affairs), and funded by BBSRC grants BB/R011311/1, BB/S006397/1, BB/X006352/1, and BB/Y513246/1 (www.ukri.org/councils/bbsrc/), a Leverhulme grant RPG-2023-001 (https://www.leverhulme.ac.uk/), and a UKRI FrontierResearch Grant EP/X024008/1 (www.ukri.org/), the Cambridge-Africa ALBORADA Research Fund (www.cambridge-africa.cam.ac.uk/), a School of Biological Sciences–Isaac Newton Trust Joint Seed Funding Award (www.newtontrust.cam.ac.uk/), and the Gatsby Charitable Foundation (www.gatsby.org.uk/). The authors have declared that the sponsors and funders played no role in the study design, data collection and analysis, decision to publish, or preparation of the manuscript.”

Please include your amended statements within your cover letter; we will change the online submission form on your behalf.

3. We note that the grant information you provided in the ‘Funding Information’ and ‘Financial Disclosure’ sections do not match.

When you resubmit, please ensure that you provide the correct grant numbers for the awards you received for your study in the ‘Funding Information’ section.

4. Thank you for stating the following financial disclosure:

“V.C.T.H. is funded by a UK Horizons: Ember Fellowship (ARIA; www.renaissancephilanthropy.org/uk-horizons/) and a Herchel Smith Postdoctoral Fellowship (www.herchelsmith.cam.ac.uk). Work on plant-parasitic nematodes at the University of Cambridge is supported by DEFRA licence 125034/359149/3 (www.gov.uk/government/organisations/department-for-environment-food-rural-affairs), and funded by BBSRC grants BB/R011311/1, BB/S006397/1, BB/X006352/1, and BB/Y513246/1 (www.ukri.org/councils/bbsrc/), a Leverhulme grant RPG-2023-001 (https://www.leverhulme.ac.uk/), and a UKRI FrontierResearch Grant EP/X024008/1 (www.ukri.org/), the Cambridge-Africa ALBORADA Research Fund (www.cambridge-africa.cam.ac.uk/), a School of Biological Sciences–Isaac Newton Trust Joint Seed Funding Award (www.newtontrust.cam.ac.uk/), and the Gatsby Charitable Foundation (www.gatsby.org.uk/). The authors have declared that the sponsors and funders played no role in the study design, data collection and analysis, decision to publish, or preparation of the manuscript.”

We note that one or more of the authors is affiliated with the funding organization, indicating the funder may have had some role in the design, data collection, analysis or preparation of your manuscript for publication; in other words, the funder played an indirect role through the participation of the co-authors. If the funding organization did not play a role in the study design, data collection and analysis, decision to publish, or preparation of the manuscript and only provided financial support in the form of authors' salaries and/or research materials, please do the following:

1. Review your statements relating to the author contributions, and ensure you have specifically and accurately indicated the role(s) that these authors had in your study. These amendments should be made in the online form.

2. Confirm in your cover letter that you agree with the following statement, and we will change the online submission form on your behalf:

“The funder provided support in the form of salaries for authors [insert relevant initials], but did not have any additional role in the study design, data collection and analysis, decision to publish, or preparation of the manuscript. The specific roles of these authors are articulated in the ‘author contributions’ section.

5. Please include captions for your Supporting Information files at the end of your manuscript, and update any in-text citations to match accordingly. Please see our Supporting Information guidelines for more information: http://journals.plos.org/plosone/s/supporting-information.

6. If the reviewer comments include a recommendation to cite specific previously published works, please review and evaluate these publications to determine whether they are relevant and should be cited. There is no requirement to cite these works unless the editor has indicated otherwise.

7. Please review your reference list to ensure that it is complete and correct. If you have cited papers that have been retracted, please include the rationale for doing so in the manuscript text, or remove these references and replace them with relevant current references. Any changes to the reference list should be mentioned in the rebuttal letter that accompanies your revised manuscript. If you need to cite a retracted article, indicate the article’s retracted status in the References list and also include a citation and full reference for the retraction notice.

Additional Editor Comments:

Revise your submission according to the comments received from two distinguished referees.

[Note: HTML markup is below. Please do not edit.]

Reviewers' comments:

Reviewer's Responses to Questions

**Comments to the Author**

1. Is the manuscript technically sound, and do the data support the conclusions?

Reviewer #1: Yes

Reviewer #2: Yes

**********

2. Has the statistical analysis been performed appropriately and rigorously?

Reviewer #1: Yes

Reviewer #2: N/A

**********

3. Have the authors made all data underlying the findings in their manuscript fully available?

The PLOS Data policy

Reviewer #1: Yes

Reviewer #2: Yes

**********

4. Is the manuscript presented in an intelligible fashion and written in standard English?

Reviewer #1: Yes

Reviewer #2: Yes

**********

Reviewer #1: The authors start from the hypothesis that Globodera might edit the DNA sequence of HYP1 in some somatic cells. They test this by attempting to distinguish rare edited somatic alleles from inherited germline variation, using Cas9-enriched long-read ONT sequencing of native DNA. If somatic editing were occurring, they would expect rare alleles carrying large, complex rearrangements of sequence motifs relative to the common germline alleles. Contrary to the hypothesis, they find only 2-4 GrHYP1 alleles among 11,378 sequences from an inbred G. rostochiensis population, and conclude that GrHYP1 is not diversified by somatic editing. They then ask the same question of G. pallida, where the updated dataset contains far fewer rare alleles than previously reported, and trace the earlier signal to nanopore basecalling error within the repetitive domain and to PCR template switching in single-worm amplicon data. Having excluded editing, they propose that the remaining germline diversity arises through error-prone homologous repair of double-strand breaks, and that genome-wide nucleotide diversity is sustained by migration between subpopulations despite small local effective population sizes.

I find the central conclusion convincing. The demonstration that two independent technical artefacts can each reproduce the exact pattern predicted by the hypothesis is valuable well beyond this system. I recommend this article for publication and I have some minor revision/questions:

In lines 129-139, the authors ask what mutations produce the complex sequence motif that defines HYP1, and they use the transgenic yeast system to conclude that erroneous homologous DNA repair of double-strand breaks are the dominant mechanism. The yeast experiment establishes that these mechanisms can generate the observed motif architecture, but not that it is the mechanism operating in Globodera. In Fig. 3., the authors establish the original construct sequence as the baseline, not the natural allelic+ diversity in G. pallida populations. Could the authors comment on whether the mutations recovered in yeast share diagnostic features with the natural GpHYP1 alleles, for instance, microhomology at rearrangement junctions or comparable size and motif-boundary distributions? Some support of this kind, or an explicit statement of the limitation would strengthen this inference.

Reviewer #2: The HYP family in PCN (potato cyst nematodes) refers to hyper-variable apoplastic effector proteins that play an essential role in how sedentary plant-parasitic nematodes establish compatible interactions with their host plants. Among HYP effectors, the HYP1 gene displays an extraordinary, unprecedented level of sequence and structural diversity, even among individual nematodes. In the submitted article, the authors tested the hypothesis that a developmentally programmed editing process exists in both species of PCN, G. rostochiensis and G. pallida. Although the results did not confirm the existence of such an editing process in plant-parasitic nematodes, they also explore other mechanisms that might explain how such a complex sequence motif structure could have arisen in the HYP1 gene.

The manuscript is well written, quite interesting (even if the main hypothesis was not validated) and I fully support its publication after the implementation of some minor comments below:

- Whilst I acknowledge that the authors present and discuss their results concisely, it seems to me that reading this article would benefit from both better structuring (subheadings) in the ‘Results/Discussion’ section and a review of what should be presented as supplementary data or within the main text. There is a great deal of supplementary data, and almost every main result or figure has a corresponding entry in the supplementary data, which readers are invited to consult. I suggest : i) providing clearer guidance to the reader regarding the supplementary information presented in the supplementary documents in relation to the figures ; ii) and also reorganizing some parts like adding the supplementary document entitled ‘HYP1 mutations observed in a transgenic yeast system’ – which contains important information – to the main text, and instead placing the paragraph in the results section (lines 116 to 128) relating to potential confounding effect in the supplementary materials (as this will not alter the main conclusion set out just before).

- Line 42-43 : Can you support by a reference the statement that free living nematodes have large census population sizes and efficient dispersal ? This was not studied in Cole & Viney, 2018 and I have the feeling that is may be an overinterpretation of the fact that free living nematodes are more aboundant in soils. I am not sure that in arable field a PPN species will not have a census population size as large of that of a free living nematode species.

- Line 79 : as the M&M is at the end, please indicate in the Results the names of the nematode populations (ie line 19 and Newton) used in this study. Qualify the Newton population as a field population maintained in laboratory rather than a « non inbred » population (there is natural inbreeding reported in many G. pallida populations, don’t think that Newton is an exception).

- Investigating mutations using the yeast system was quite interesting but if I am correct yeast is a good model with great functional conservation accross animals for nucleotide excision or mismatch repair but I don’t know if this holds true also for recombinational mutations. May be a point of discussion or a paragraph to add in the introduction in relation to this part of the results.

- Figure 4D : I have not understood the interest of Figure 4D and have not seen any corresponding text in lines 152-160.

- Lines 161 - 179: I am not convinced of the value of this paragraph and I think it should be deleted. Indeed, it adds nothing new: even if the estimate of Ne differed from that already published for G. pallida, it would be impossible to compare them, as the value obtained by the authors is closely linked to the population’s rearing conditions in the laboratory. Furthermore, as the authors themselves mention, their estimate of Ne could be overestimated, as many parameters of the ideal population, as defined in the Wright–Fisher model, are / can be biased (an ideal population is characterised by the absence of migration, mutation, selection and generational overlap, by equal sex ratios, a constant size from one generation to the next, and random mating).

**********

what does this mean?). If published, this will include your full peer review and any attached files.

If you choose “no”, your identity will remain anonymous but your review may still be made public.

**Do you want your identity to be public for this peer review?**  For information about this choice, including consent withdrawal, please see our Privacy Policy

Reviewer #1: No

Reviewer #2: No

**********

[NOTE: If reviewer comments were submitted as an attachment file, they will be attached to this email and accessible via the submission site. Please log into your account, locate the manuscript record, and check for the action link "View Attachments". If this link does not appear, there are no attachment files.]

To ensure your figures meet our technical requirements, please review our figure guidelines: https://journals.plos.org/plosone/s/figures

You may also use PLOS’s free figure tool, NAAS, to help you prepare publication quality figures: https://journals.plos.org/plosone/s/figures#loc-tools-for-figure-preparation.

NAAS will assess whether your figures meet our technical requirements by comparing each figure against our figure specifications.

# Author comment: Author response to Decision Letter 1

**DOI:** 10.1371/journal.pone.0358232.r002

27 Aug 2026

Please see the attached "Response to Reviewers" document

Attachment Submitted filename: Response to Reviewers.pdf

# Editor report: Decision Letter 1

**DOI:** 10.1371/journal.pone.0358232.r003

**Authors:** Tushar Kanti Dutta

30 Aug 2026

Diversity at the HYP1 locus in potato cyst nematodes does not result from developmentally-programmed somatic mutations

PONE-D-26-37792R1

Dear Dr. Hanlon,

We’re pleased to inform you that your manuscript has been judged scientifically suitable for publication and will be formally accepted for publication once it meets all outstanding technical requirements.

Within one week, you’ll receive an e-mail detailing the required amendments. When these have been addressed, you’ll receive a formal acceptance letter and your manuscript will be scheduled for publication.

An invoice will be generated when your article is formally accepted. Please note, if your institution has a publishing partnership with PLOS and your article meets the relevant criteria, all or part of your publication costs will be covered. Please make sure your user information is up-to-date by logging into Editorial Manager at Editorial Manager® and clicking the ‘Update My Information' link at the top of the page. For questions related to billing, please contact billing support.

If your institution or institutions have a press office, please notify them about your upcoming paper to help maximize its impact. If they’ll be preparing press materials, please inform our press team as soon as possible -- no later than 48 hours after receiving the formal acceptance. Your manuscript will remain under strict press embargo until 2 pm Eastern Time on the date of publication. For more information, please contact onepress@plos.org.

Kind regards,

Tushar Kanti Dutta, Ph.D.

Academic Editor

PLOS One

Additional Editor Comments (optional):

Reviewers' comments:

# Editor report: Acceptance letter

**DOI:** 10.1371/journal.pone.0358232.r004

**Authors:** Tushar Kanti Dutta

PONE-D-26-37792R1

PLOS One

Dear Dr. Hanlon,

I'm pleased to inform you that your manuscript has been deemed suitable for publication in PLOS One. Congratulations! Your manuscript is now being handed over to our production team.

At this stage, our production department will prepare your paper for publication. This includes ensuring the following:

* All references, tables, and figures are properly cited

* All relevant supporting information is included in the manuscript submission,

* There are no issues that prevent the paper from being properly typeset

You will receive further instructions from the production team, including instructions on how to review your proof when it is ready. Please keep in mind that we are working through a large volume of accepted articles, so please give us a few days to review your paper and let you know the next and final steps.

Lastly, if your institution or institutions have a press office, please let them know about your upcoming paper now to help maximize its impact. If they'll be preparing press materials, please inform our press team within the next 48 hours. Your manuscript will remain under strict press embargo until 2 pm Eastern Time on the date of publication. For more information, please contact onepress@plos.org.

You will receive an invoice from PLOS for your publication fee after your manuscript has reached the completed accept phase. If you receive an email requesting payment before acceptance or for any other service, this may be a phishing scheme. Learn how to identify phishing emails and protect your accounts at https://explore.plos.org/phishing.

If we can help with anything else, please email us at customercare@plos.org.

Thank you for submitting your work to PLOS One and supporting open access.

Kind regards,

PLOS One Editorial Office Staff

on behalf of

Dr. Tushar Kanti Dutta

Academic Editor

PLOS One
